# Epigallocatechin-3-Gallate Therapeutic Potential in Cancer: Mechanism of Action and Clinical Implications

**DOI:** 10.3390/molecules28135246

**Published:** 2023-07-06

**Authors:** Mateusz Kciuk, Manzar Alam, Nemat Ali, Summya Rashid, Pola Głowacka, Rajamanikandan Sundaraj, Ismail Celik, Esam Bashir Yahya, Amit Dubey, Enfale Zerroug, Renata Kontek

**Affiliations:** 1 Department of Molecular Biotechnology and Genetics, University of Lodz, Banacha Street 12/16, 90-237 Lodz, Poland; mateusz.kciuk@edu.uni.lodz.pl (M.K.); renata.kontek@biol.uni.lodz.pl (R.K.); 2 Doctoral School of Exact and Natural Sciences, University of Lodz, Banacha Street 12/16, 90-237 Lodz, Poland; 3 Centre for Interdisciplinary Research in Basic Sciences, Jamia Millia Islamia, New Delhi 110025, India; manzar987@gmail.com; 4 Department of Pharmacology and Toxicology, College of Pharmacy, King Saud University, Riyadh 11451, Saudi Arabia; nali1@ksu.edu.sa; 5 Department of Pharmacology & Toxicology, College of Pharmacy, Prince Sattam Bin Abdulaziz University, P.O. Box 173, Al-Kharj 11942, Saudi Arabia; 6 Department of Medical Biochemistry, Medical University of Lodz, Mazowiecka 6/8, 90-001 Lodz, Poland; pola.glowacka@edu.uni.lodz.pl; 7 Doctoral School of Medical University of Lodz, Hallera 1 Square, 90-700 Lodz, Poland; 8 Department of Biochemistry, Centre for Drug Discovery, Karpagam Academy of Higher Education, Coimbatore 641021, India; mani.bioinfor@gmail.com; 9 Department of Pharmaceutical Chemistry, Faculty of Pharmacy, Erciyes University, Kayseri 38280, Turkey; ismailcelik@erciyes.edu.tr; 10 Bioprocess Technology Division, School of Industrial Technology, Universiti Sains Malaysia, Penang 11800, Malaysia; essam912013@gmail.com; 11 Computational Chemistry and Drug Discovery Division, Quanta Calculus, Greater Noida 201310, India; ameetbioinfo@gmail.com; 12 Department of Pharmacology, Saveetha Institute of Medical and Technical Sciences, Saveetha Dental College and Hospital, Chennai 600077, India; 13 LMCE Laboratory, Group of Computational and Pharmaceutical Chemistry, University of Biskra, Biskra 07000, Algeria; anfelzerroug220@gmail.com

**Keywords:** bioavailability, clinical trials, epigallocatechin gallate, signaling pathways

## Abstract

Cellular signaling pathways involved in the maintenance of the equilibrium between cell proliferation and apoptosis have emerged as rational targets that can be exploited in the prevention and treatment of cancer. Epigallocatechin-3-gallate (EGCG) is the most abundant phenolic compound found in green tea. It has been shown to regulate multiple crucial cellular signaling pathways, including those mediated by EGFR, JAK-STAT, MAPKs, NF-κB, PI3K-AKT-mTOR, and others. Deregulation of the abovementioned pathways is involved in the pathophysiology of cancer. It has been demonstrated that EGCG may exert anti-proliferative, anti-inflammatory, and apoptosis-inducing effects or induce epigenetic changes. Furthermore, preclinical and clinical studies suggest that EGCG may be used in the treatment of numerous disorders, including cancer. This review aims to summarize the existing knowledge regarding the biological properties of EGCG, especially in the context of cancer treatment and prophylaxis.

## 1. Introduction

**Figure 1:**
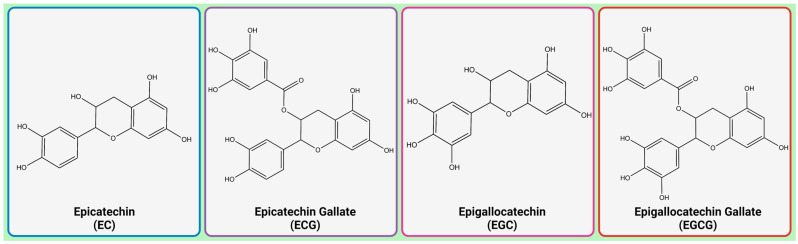
Chemical structure of epicatechin (EC), epicatechin-3-gallate (ECG), epigallocatechin (EGC), and epigallocatechin-3-gallate (EGCG). Created with BioRender.com, accessed on 29 March 2023.

Green tea (*Camellia sinensis*) consumption is known for its health benefits [1,2]. The majority of green tea’s positive effects on human health can be attributed to the high polyphenol and flavonoid content of the beverage. Catechins, which are the primary flavonoids found in green tea, account for about 30–40% of the solid components of this plant. Epicatechin (EC), epigallocatechin (EGC), epicatechin-3-gallate (ECG), and epigallocatechin-3-gallate (EGCG) are the primary catechins found in tea (Figure 1) [3].

EGCG has gained particular scientific interest in the past decade due to its numerous health benefits resulting from its antioxidant, anti-inflammatory, anti-fibrotic, and anti-cancer properties [4]. EGCG is consumed mainly via brew drinking. One cup of green tea contains approximately 177 mg of EGCG [5,6]. Therapeutic advantages of green tea consumption have been observed in inflammatory diseases and several types of cancer [7,8,9]. The anti-tumor activity of EGCG has been linked to the inhibition of the proteins involved in cellular signaling pathways that are frequently disrupted in cancer. These include the protein networks mediated by epidermal-growth-factor receptor (EGFR) [10,11,12,13], tyrosine-protein kinase JAK (JAK), signal transducer and activator of transcription (STAT) (JAK/STAT pathway) [14,15,16], phosphoinositide-3-kinase (PI3K), RAC-alpha serine/threonine-protein kinase (AKT), serine/threonine-protein kinase mTOR (mTOR) (PI3K/AKT/mTOR pathway) [10,17,18], and mitogen-activated protein kinases (MAPK/ERK pathways) [19,20]. Moreover, EGCG exhibits antioxidant, anti-inflammatory, and anti-angiogenic effects [4] and may promote both caspase-dependent [21,22,23] and caspase-independent cell death [24]. The PubMed database (https://pubmed.ncbi.nlm.nih.gov/; accessed on 29 March 2023) was searched using the combination of the following keywords: “epigallocatechin 3-gallate”, ”EGCG”, “bioavailability”, “epigenetics”, and “signaling pathways”. Additionally, the “clinical trial” option of the PubMed search was chosen to include all the reported clinical trials on EGCG. The rationale behind this study was to gather information regarding the molecular mode of the anticancer activity of EGCG and to discuss the critical concerns associated with the use of EGCG in clinical settings including its bioavailability and toxicity. We aimed to answer the following questions: (a) What are the major pathways associated with the anticancer activity of EGCG? (b) Does EGCG exert its activity through epigenetic influence? (c) What are the emerging targets of EGCG? (d) What evidence has been provided by epidemiological studies and clinical studies of EGCG? (e) What is and what influences the bioavailability of EGCG? (f) Is EGCG use safe and what doses are tolerable in humans? 

## 2. Mechanism of Anticancer Activity of EGCG

Medicinal plants have been used for centuries in the prevention and management of various diseases. Natural products (NPs) constitute sources of bioactive molecules that can be employed in the treatment of human conditions or can be modified to generate new, more efficient, and less toxic drugs with improved bioavailability [25,26,27]. Their therapeutic properties are notably valued in oncology. It is believed that approximately 60% of all oncopharmaceuticals currently available on the market have been designed based on plant-derived compounds [28,29,30]. In the meantime, the multidimensional action mechanisms of these compounds are being discovered along with their abilities to concurrently impact multiple oncogenic signaling pathways. This translates into the capability of cells to either proliferate, migrate, metastasize, or bypass apoptotic cell death. Because of the variety of their properties, natural compounds can be utilized as either direct cytotoxic agents or chemotherapy adjuvants. This allows them to support anticancer activity, overcome the therapy resistance of cancer cells, and enhance the repair processes of normal cells. Furthermore, compared to synthetically obtained compounds, the costly and time-consuming selection of leading structures is avoided, and efforts can be directed at synthesizing and testing new derivatives to obtain better pharmacological responses [31,32].

EGCG’s multidirectional anti-tumor effects have been confirmed in multiple in vitro and in vivo studies investigating the effects of this polyphenol in cancer cells originating from different tissues, including bladder cancer [33,34,35,36,37,38,39,40,41,42,43], breast cancer [21,44,45,46,47,48,49,50,51,52], cervical cancer [53,54,55,56,57,58,59], colorectal cancer [60,61,62,63,64,65], gastric [66,67,68,69], liver [70,71,72,73], lung cancer [11,22,74,75,76], and head and neck cancers [77,78,79].

### 2.1. Antioxidant Properties

In aerobic organisms, reactive oxygen species (ROS) are produced in the electron transport chain (ETC) or via the activity of catabolic oxidases, anabolic metabolism, and peroxisomal metabolism [80,81,82]. At low levels, ROS operate as cellular messengers in redox-signaling events [83]. However, an excessive production of ROS can lead to DNA damage in the form of roughly 100 distinct oxidative base lesions and 2-deoxyribose alterations [84,85,86]. ROS generation is normally controlled in cells by three distinct mechanisms: the restriction of respiration in the mitochondrial compartment, the complexation of DNA with protective histones, and the elimination of excess ROS by antioxidant enzymes [87,88,89,90,91]. Superoxide radicals, hydrogen peroxide, and hydroxyl radicals are the most prominent ROS species generated in cells [92,93]. EGCG was found to have a free-radical-scavenging activity that allows it to trap hydroxyl radicals and superoxide anions and thus suppress and terminate the free radical chain reaction that occurs during lipid peroxidation. The oxidative-stress-relieving properties of EGCG are achieved via the inhibition of pro-oxidant enzymes such as NADPH oxidase, the activation of antioxidant systems such as superoxide dismutase, catalase, or glutathione, and by decreasing the generation of nitric oxide metabolites by inducible nitric oxide synthase (iNOS) [94].

It would appear that EGCG is the most active of all the catechins, having an antioxidant activity that is 25 to 100 times stronger than that of vitamins C and E [95]. The antioxidant properties of this flavonoid have been extensively described in the literature [3,96,97] and will not be discussed here in detail. Attempts have been undertaken to further increase the antioxidant capacities of EGCG and enhance its lipophilicity. It was found that acetylation of this compound may boost its antioxidant properties [98].

### 2.2. Suppression of Inflammation

Prolonged chronic inflammation is considered a hallmark of tumor development. It has been found to promote all stages of tumorigenesis. In the process of inflammation, a large number of immune cells, including neutrophils, monocytes, and macrophages, are recruited to the sites of action and secrete various molecules (cytokines and enzymes) that regulate the process. These include tumor necrosis factor α (TNF-α), interleukins (IL-1β, IL-4), matrix metalloproteinases (MMPs), and cyclooxygenases (COXs). An increased expression of many of these inflammatory mediators has been linked to worse prognosis of tumor-bearing patients [99,100,101,102]. The results of multiple studies have demonstrated that EGCG modulates the expression of inflammation-associated genes and proteins such as TNF-α, IL-1β, and MMPs [8]. Here, we focus on the targets of EGCG that exhibit a role in inflammation in the context involving cancer development.

TNF-α is a pleiotropic cytokine that plays an important part in apoptosis and cell survival in addition to its canonical roles in inflammation and immunity. TNFα earned its acronym because of its ability to fight tumors; however, recent research has linked it to numerous other disorders. TNF-α modulates versatile cellular functions as a result of its contact with TNF receptors 1 and 2 (TNFR-1 and TNFR-2). This association stimulates multiple signal transduction pathways including nuclear factor kappa B (NF-κB) and c-Jun N-terminal kinase (JNK), which in turn lead to multiple context-dependent outcomes including apoptosis, necrosis, angiogenesis, immune cell activation, differentiation, and cell migration. While the continuous activation of JNK is associated with cell death, NF-κB is a significant anti-apoptotic factor that promotes cell survival. It should therefore not come as a surprise that TNFα, in a manner that is dependent on the setting, can demonstrate both pro- and antitumoral actions. Hence, the regulation of the activity of the TNFR pathway opens up a wide variety of therapeutic avenues for the treatment of cancer [103,104,105,106,107]. Multiple studies have found that EGCG can suppress TNFα expression and inhibit the activation of TNFα-mediated pathways. However, the studies were not conducted in the context of cancer. For example, EGCG was able to significantly inhibit the expression of monocyte chemoattractant protein-1 (MCP-1) that was stimulated by TNF-α in human umbilical vein endothelial cells (HUVECs). EGCG appeared to inhibit the TNFα-induced activation of NF-κB. In addition, the suppression of 67-kD laminin receptor (67LR) played a significant role in reducing the synthesis of MCP-1 following EGCG treatment [108]. The generation of TNF-α, IL-6, and IL-8 was decreased in human mast cells (HMC-1 cell line) by EGCG. This was accomplished by decreasing the intracellular Ca^2+^ levels as well as via the repression of the ERK1/2- and NF-κB-mediated signaling pathways [109].

Cyclooxygenases (COXs) are enzymes that are important for the metabolic conversion of arachidonic acid to prostaglandins, particularly PGE2, which is a significant mediator of both inflammation and angiogenesis. COX-1 and COX-2 are two different isoforms of the enzyme. Both are found in cells as membrane-bound proteins, which constitute their integral part. Inside the cell, they are typically found on the luminal side of the endoplasmic reticulum and the nuclear envelope. COX-1 is referred to as a housekeeping enzyme since it is necessary for the upkeep of baseline levels of prostaglandins. In contrast, COX-2 is not often detectable in the healthy tissues and organs of the body but is highly inducible, and its expression can be rapidly increased in reaction to a wide range of proinflammatory agents, such as cytokines or mitogens [110]. COX-2 is commonly over-expressed in a wide variety of malignancies. This enzyme plays a pleiotropic and multidimensional role in the initiation or development of cancer as well as its resistance to chemo- and radiotherapy. Cancer-associated fibroblasts (CAFs) and tumor-associated macrophages (TAMs) that are present in the tumor microenvironment (TME), contribute to the production of COX-2, which in the latter step is released into the tumor setting [111]. It appears that the constitutive expression of COX-2 and the prolonged synthesis of PGE2 play predominant roles in the onset and development of cancer. PGE2 is capable of mediating these effects through a wide variety of signaling pathways, such as the activation of the vascular endothelial growth factor receptor (VEGFR) pathway, which can lead to an increased proliferation, metastatic potential, and angiogenesis of cells and an increased expression of protooncogenes such as BCL-2 [110]. This topic has been extensively discussed by previous authors [110,111,112,113,114,115].

It was demonstrated that EGCG had a strong inhibitory effect on the constitutive overexpression of COX-2 in colon cancer [116] and prostate cancer cells [117]. It was found that EGCG concurrently decreased the activity of the ERK1/2 and AKT pathways and reduced COX-2 promoter activity via its suppressive effect on NF-κB action [116]. Moreover, it was found that a combination of EGCG and COX-2 inhibitor (NS-398) exhibited a synergic anticancer activity, which was indicated by an elevated apoptotic response (increase in the expression of pro-apoptotic BAX protein, pro-caspase-6, and pro-caspase-9 and the cleavage of poly(ADP)ribose polymerase (PARP)) and the inhibition of NF-κB activity [118]. The selective inhibition of COX2 was also observed in EGCG-treated prostate cancer cells (LNCaP and PC-3) [117]. EGCG was found to inhibit early- but not late-stage prostate cancer in vivo, as evidenced by the suppression of cell proliferation and the induction of apoptosis. Furthermore, a decrease in the protein levels of androgen receptor (AR), insulin-like growth factor-1 (IGF-1), IGF-1 receptor (IGF-1R), phosphorylated ERK1 and ERK2, COX-2, and iNOS were observed [119].

Nitric oxide (NO) is a pleiotropic mediator that plays an essential role in a wide variety of biological processes. Some of these functions include vasodilatation or macrophage-mediated immune protection. NOS activity has been found in tumor cells with a variety of different histogenetic origins. This activity has been linked to the rate of tumor proliferation and the expression of significant signaling components that are associated with the development of cancer, such as the estrogen receptor. It would appear that a high production of NO (for example, that generated by activated macrophages) would be cytotoxic or cytostatic for tumor cells, but low levels of NO may have the reverse effect and encourage the growth of tumors. In addition, NO can affect the DNA repair mechanisms of tumors through the up-regulation of genes such as cellular tumor antigen p53 (TP53), PARP, and DNA-dependent protein kinase (DNA-PK) [120,121,122]. It was discovered that EGCG lowers NO generation. This effect may be caused by EGCG through one of two mechanisms: either a decrease in iNOS gene expression or the suppression of enzyme function [123,124].

NF-κB plays a role not only in the control of cellular immunity, inflammation, and stress but also cell differentiation, proliferation, and apoptosis. NF-κB activity and function are often altered in both solid and hematologic malignancies [125]. NF-κB can be activated by a variety of stimuli, including cytokines (such as TNF-α and IL-1β), growth factors (such as EGF), ultraviolet and ionizing radiation, ROS, and DNA damage. The common feature of these triggers is that they ultimately result in the activation of a large cytoplasmic protein complex known as the inhibitor of the κB (IκB) kinase (IKK) complex [126]. NF-κB was found to induce the expression of anti-apoptotic factors such as caspase-8 inhibitor cellular FLICE-like inhibitory protein (FLIP), the inhibitor of the apoptosis proteins c-IAP1/2 and E3 ubiquitin-protein ligase XIAP, or members of the BCL2 family of apoptosis regulators. Moreover, it controls the expression of many angiogenic factors and metastasis-associated proteins including VEGF or MMPs and glucose-metabolism-associated proteins, including glucose transporters (GLUTs) [126].

**Figure 2:**
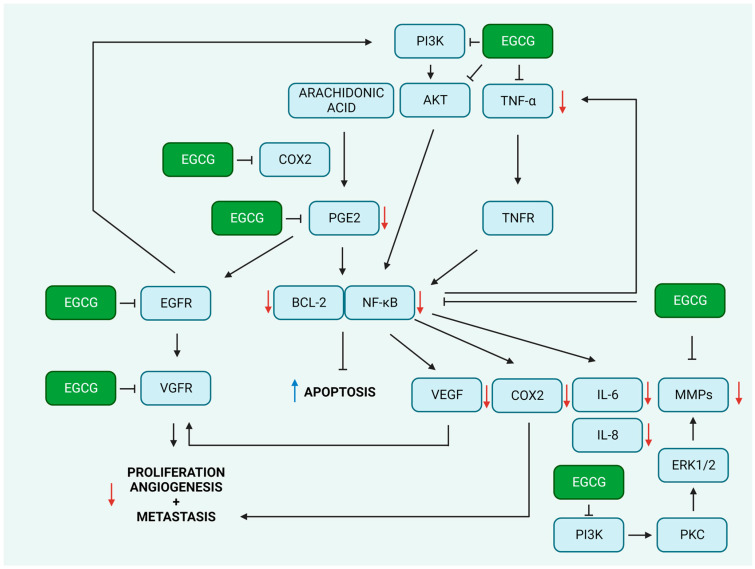
The molecular basis of the anticancer mechanism of action of epigallocatechin-3-gallate (EGCG) related to the suppression of inflammation. Cyclooxygenase 2 enzyme (COX2) catalyzes the conversion of arachidonic acid to prostaglandins (PGEs), including PGE2. PGE2 stimulates the expression of anti-apoptotic BCL-2 protein and the nuclear factor NF-kappa-B (NF-κB)-mediated gene expression of vascular endothelial growth factor, interleukins (IL-6 and -8), matrix metalloproteinases (MMPs), and COX2. EGCG was shown to act as a COX2, PGE2, NFκB, and MMPs inhibitor, suppressing the angiogenesis and metastasis of cancer cells and enhancing apoptosis induction. Additionally, EGCG may work as phosphoinositide-3-kinase/RAC-alpha serine/threonine-protein kinase (PI3K/AKT), epithelial growth factor (EGFR), and vascular endothelia growth factor, which control NF-κB and MMPs activity and expression and confer enhanced proliferation, angiogenesis, and metastasis. Furthermore, EGCG treatment may down-regulate the expression of tumor necrosis factor α (TNF-α) and suppress the activation of tumor necrosis factor receptor (TNFR) signaling. For full protein names, see the abbreviations section. Red arrows indicate down-regulation of the protein expression or its inhibition. Created with BioRender.com, accessed on 29 March 2023.

MMPs belong to the family of zinc-dependent extracellular matrix (ECM) remodeling enzymes that are capable of degrading practically every component of the ECM. The breakdown of the ECM is essential for cancer cells to spread from the primary tumor site to other sites of the body. The degradation of ECM by MMPs not only promotes tumor invasion but also alters the behavior of tumor cells and contributes to the progression of cancer, as previously reviewed [127,128,129]. Several studies have indicated that EGCG may inhibit the activity and expression of MMPs. In line with this evidence, a molecular docking study confirmed these inhibitory effects. In their in silico studies, Chowdhury et al. showed that EGCG and ECG exhibit superior inhibitory properties towards proMMP2 compared with MMP2 [130]. Furthermore, Sarkar et al. employed a molecular docking approach and revealed that EGCG may inhibit proMMP9 and MMP9. The enhanced interaction between EGCG/MMPs compared to EC and EGC may be attributed to the presence of a gallate group in the EGCG structure. In particular, EGCG’s galloyl moiety binds to Phe201, His401, Glu402, His411, and Pro421 residues in the MMP-9 structure. The inclusion of a galloyl residue in EGCG and ECG contributes to a binding affinity that is approximately 1.5-fold higher than that of EC and EGC [131]. These findings are consistent with earlier zymographic studies conducted by Maeda-Yamamoto et al. [132]. Furthermore, it was shown that EGCG inhibited the migration and invasion of T24 human bladder cancer cells through the inhibition of the PI3K/AKT pathway that further conferred the inactivation of NF-κB and the down-regulation of MMP-9 expression, ultimately limiting the metastatic potential of the cells [40,42]. A similar effect was observed for SW780 bladder cancer cells, where EGCG down-regulated the expression of NF-κB and MMP-9 and triggered the apoptosis of cancer cells [33]. EGCG was tested for synergic anticancer effects with a commonly used anticancer agent—doxorubicin (DOX). EGCG was found to boost DOX’s ability to induce apoptosis. In addition, EGCG enhanced DOX’s ability to prevent the migration of bladder cancer cells. According to the findings of mechanistic investigations, the combination of DOX and EGCG suppressed the expression of phosphorylated NF-κB and E3 ubiquitin-protein ligase Mdm2 (MDM2) while simultaneously elevating the expression of TP53 in tumor cells in vivo [34]. Similar observations were made in breast cancer cells, where EGCG suppressed the activation of hypoxia-inducible factor 1-alpha (HIF-1α) and NFκB as well as VEGF expression [47]. Cervical cancer cells (HeLa cell line) treated with EGCG and black tea polyphenol theaflavins displayed a significant inhibition of proliferation, an increase in the number of cells in the sub-G1 cell-cycle phase, and a reduction in the mitochondrial membrane potential (MMP) alongside an increase in the production of ROS, an overexpression of TP53, a change in the BAX/BCL-2 ratio, a release of cytochrome-c, procaspase-3, -9 activation, and PARP cleavage. In addition, both EGCG and theaflavins were able to suppress the activation of AKT and NF-κB, which resulted in the down-regulation of COX2 and cyclin D [55]. Furthermore, EGCG may down-regulate the expression of MMP-9 and therefore decrease the invasive potential of HeLa cells [56]. The anti-inflammatory potential of EGCG is presented in Figure 2.

### 2.3. Modulation of Epigenetic Targets

The recent decades of cancer research have revealed that epigenetic mechanisms play crucial roles in both the onset and development of tumors. Genome-wide DNA hypomethylation and site-specific DNA hypermethylation, histone modifications, micro RNAs, long non-coding RNAs, and nucleosome remodeling dysregulations are all common epigenetic alterations observed in cancer. An evident therapeutic potential for reversing these epigenetic changes has been demonstrated in a variety of lymphomas, and similar results are beginning to appear in the treatment of solid tumors [133,134]. Thereby, the use of selective agents (termed epi-drugs) to target the cancer epigenome or cancer epigenetic dysregulation is an emerging and exciting strategy for cancer prevention and treatment [135].

DNA methylation is one of the epigenetic regulatory mechanisms that was discovered first and has received the most attention in cancer research. DNA methyltransferases (DNMTs) and DNA demethylases are primarily responsible for the cooperative regulation that determines the level of methylation across the genome. Using S-adenosylmethionine (SAM) as a donor, DNMTs add a methyl group (CH3) to the C5 position of the cytosine ring in cytosine-phosphate-guanine (CpG) islands located in the promoters of genes [136]. DNMTs such as DNMT1, DNMT3A, and DNMT3B write and control patterns of DNA methylation in mammalian cells. DNMT1 is typically responsible for the proper methylation pattern on the daughter strand copies produced from parent DNA during the replication process. In contrast, de novo methyltransferases 3A and 3B are essential in the process of genomic imprinting during embryogenesis and germ cell development. The overexpression of DNMTs has been linked with carcinogenesis and poor disease prognosis [137,138,139,140]. Moreover, many tumors exhibit somatic mutations in DNMTs, which significantly contribute to malignant transformation [141].

A time-dependent inhibition of the enzymatic activity of DNMTs was observed in HeLa cells treated with EGCG. The expression of DNMT3B was also found to decrease over time in EGCG-treated HeLa cells. Thereby, it was suggested that EGCG may serve as a potential epigenetic modifier by altering the methylation status of genes encoding retinoic acid receptor beta (RARβ), cadherin 1 (CDH1), and death-associated protein kinase 1 (DAPK1) tumor suppressors to reactivate their expression [57]. RARβ is a nuclear receptor that is crucial in cell differentiation, apoptosis, and proliferation. Research has indicated that RARβ is commonly repressed or silenced in multiple cancer types, and its loss has been linked with resistance to therapy and tumor progression. RARβ can halt the cell cycle at the G1/S phase [142,143]. CDH1 (E-cadherin), is a molecule crucial for cell adhesion, polarity, and epithelial tissue integrity. Mutations or losses of CDH1 are frequently observed in cancer and contribute to the invasion and metastasis of tumors [144]. DAPK1 is a protein kinase that regulates tumor suppression, autophagy, and apoptosis. DAPK1 is frequently down-regulated, which promotes tumor formation, metastasis, and resistance to chemotherapy [145].

Human epidermoid carcinoma A431 cells treated with EGCG showed a dose-dependent reduction in global DNA methylation. DNMT activity, mRNA, and protein levels for DNMT1, DNMT3a, and DNMT3b were all reduced by EGCG with a concomitant decrease in 5-methylcytosine abundance. Additionally, EGCG lowered histone deacetylase activity (HDAC) and increased acetylated lysine levels in histone H3 (H3K9 and H3K14) and histone H4 (H4K5, H4K12, and H4K16). In most cases, these acetylation events contribute to the open chromatin state, which makes DNA more accessible to transcription factors and other proteins that regulate gene expression. Analogously, an observed decrease in methylated H3K9 leads to an open chromatin state. P16INK4a and P21/P27 tumor suppressor genes that had been silenced were re-expressed in response to EGCG treatment. The primary function of p16INK4a is to suppress the activities of CDK4/6, which controls the cell cycle by facilitating the transition from the G1 phase to the S phase. In contrast, P21 is a protein that serves as a regulator of the cell cycle as well as cellular responses to DNA damage. The primary function of P21 is to regulate the G1/S checkpoint, which is the moment of the cell cycle at which the cell decides whether to continue the cell division or enter a non-dividing state [146].

Histone and non-histone proteins are frequently modified post-translationally with reversible acetylation. Two classes of enzymes, histone acetyltransferases (HATs) and HDACs, work against each other to acetylate and deacetylate proteins, respectively. Both an abnormal expression of HDACs and aberrant protein acetylation, especially histones, have been linked to cancer. Therefore, HDACs have become interesting therapeutic targets for cancer [147]. EC, EGC, and EGCG were indicated as HAT inhibitors in AR-dependent prostate cancer cells. EGCG showed the strongest inhibitory potential among the catechins studied and suppressed AR acetylation and its translocation from the cytoplasm to the nucleus [148]. As nuclear factor NF-kappa-B p65 subunit (P65) acetylation by P300/CBP is required for NF-κB activation, decreasing P65 acetylation may constitute a useful therapeutic approach for the alleviation of persistent inflammation. EGCG was discovered to block HATs and, as a result, reduce the P300-dependent acetylation of P65 in vitro and in vivo, elevate cytosolic IκBα levels, and inhibit NF-κB activation in response to TNF-α. In addition, EGCG therapy suppressed P65 acetylation and NF-κB target gene expression in response to multiple stressors. The necessity of a proper balance between HATs and HDACs in the NF-κB-mediated inflammatory signaling pathway was further highlighted by the observation that EGCG inhibited p300 binding to the promoter region of the *IL-6* gene and resulted in an enhanced recruitment of HDAC3. These findings suggest that EGCG can be helpful in the prevention of EBV-induced B lymphocyte transformation [149]. Furthermore, the study of Borutinskaitė et al. showed that EGCG was able to suppress the proliferation of NB4 and HL-60 cells, induce apoptosis, and stimulate the gene expression of proteins that are involved in the cell cycle arrest and the differentiation of cells (e.g., P27, histone acetyltransferase KAT2B (PCAF), and CCAAT/enhancer-binding proteins (C/EBPa and C/EBPE)). EGCG down-regulated the expression of DNMT1 and HDAC1/2 as well as histone methyltransferase G9a and polycomb-repressive complex 2 (PRC2), which are involved in chromatin remodeling. These findings suggested that EGCG could be an effective epigenetic therapeutic agent that could be used in the treatment of acute promyelocytic leukemia (APL) [150].

The abnormal epigenetic silencing of the tissue inhibitor of the matrix metalloproteinase-3 (TIMP-3)-encoding gene, which functions to negatively limit the activity of MMPs, has been linked to the progression and metastasis of breast and prostate cancers. EGCG was shown to mediate the epigenetic elevation of TIMP-3 levels and play a critical role in the regulation of the enzymatic activity of MMP-2 and MMP-9 in breast cancer cells. This was associated with a reduction in the levels of enhancers of zeste homolog 2 (EZH2) and class I (HDAC1). EZH2 works as a histone methyltransferase that catalyzes the addition of a methyl group to lysine 27 of histone H3 (H3K27) and functions as a part of the catalytic subunit of PRC2. It was shown that transcriptional activation of TIMP-3 was related to decreased EZH2 localization and H3K27 trimethylation enrichment at the TIMP-3 promoter, along with a simultaneous rise in histone H3K9/18 acetylation. H3K27 trimethylation introduced as a PRC2 works as a repressive mark that is associated with transcriptional silencing and the formation of heterochromatin. Additionally, it provides a binding site for proteins that contain a chromodomain, such as polycomb group (PcG) proteins. In contrast, H3K9/18 acetylation is associated with an open chromatin state [151,152].

**Figure 3:**
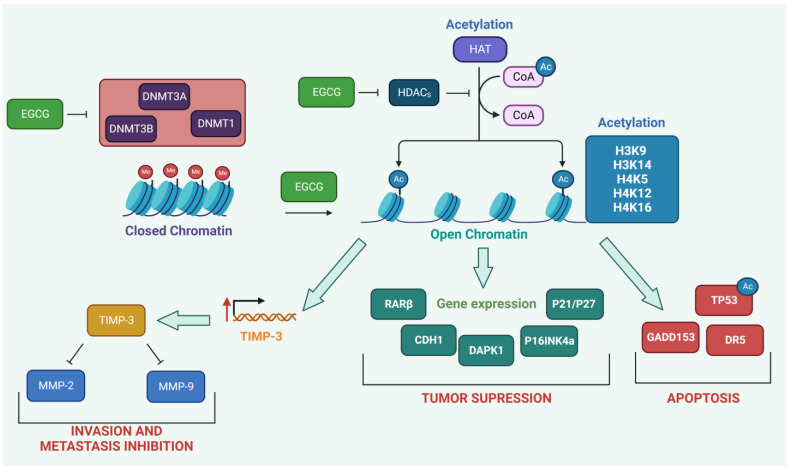
The modulation of epigenetic targets by epigallocatechin-3-gallate (EGCG). Methylation of CpG islands and histone proteins works as a mark that confers the closed chromatin formation and transcriptional suppression of genes encoding proteins involved in tumor suppression, invasion and metastasis inhibition, and apoptosis induction. EGCG down-regulates and inhibits the activity of DNA methyltransferases (DNMT1, DNMT3A, and 3B) that introduce methyl groups to DNA. Moreover, EGCG was shown to inhibit the activity of histone deacetylases (HDACs) that counteract the introduction of acetyl groups to histone proteins via histone acetyltransferases (HATs). The introduction of acetyl groups to histone proteins (H3K9, H3K14, H4K5, H4K12, and H4K16) contributes to the open chromatin state and elevated expression of tumor suppressor proteins (CDH1, DAPK1, P12, P16INK4a, P27, and RARβ), proteins involved in apoptosis induction and cell death control (DR5, GAD153, and TP53), and TIMP-3, which inhibits the proteolytic activity of MMP-2 and MMP-9. For full protein names, see the abbreviations section. Created with BioRender.com, accessed on 29 March 2023.

A synergistic induction of apoptosis and an up-regulated expression of growth arrest and DNA-damage-inducible gene 153 (*GADD153*), death receptor 5 (DR5), and P21 were shown following the treatment of human lung cancer cells (PC-9 cell line) with a combination of EGCG and synthetic retinoid (Am80). Despite no change in acetylation levels in histones H3 or H4, the combination boosted acetylated TP53 and acetylated tubulin levels through a decrease in HDAC activity in the cytosol fraction. Additionally, the combination decreased the protein levels of HDAC4, −5, and −6 by 20% to 80%. By inhibiting HDAC4, −5, and −6, EGCG and Am80 altered acetylation levels in nonhistone proteins and induced the cell death of cancer cells [153]. The modulation of epigenetic targets by EGCG is shown in Figure 3.

### 2.4. Modulation of Cellular Signaling Molecules by EGCG

#### 2.4.1. EGFR Pathway

The EGFR pathway plays a major role in epithelial cell proliferation, survival, and differentiation as well as autophagy and cell metabolism [154,155]. The disruption of its function often leads to neoplasia and therapy escape mechanisms [156,157]. In several human cancers, including glioblastoma, colorectal cancer, breast cancer, and lung cancer, the *EGFR* gene is often amplified and mutated and the EGFR protein is overexpressed, leading to chronic receptor activation, excessive signaling, and the promotion of neoplastic transformation [156,158,159].

Normally, EGFR is situated on the cell surface. While not activated by one of seven possible ligands, it remains a monomer with minimal kinase activity [160,161]. After ligand activation, EGFR can either create a homodimer or a heterodimer with other receptors from the ErbB/HER tyrosine kinase receptor family, with the second option usually generating a stronger signal transduction and more oncogenic potential [162,163]. These conformational changes lead to the autophosphorylation of tyrosine residues in the receptor tail located in the cytoplasm, triggering a signaling cascade [163]. The cellular response is dependent on the specific ligand that has activated EGFR. The reaction usually involves the activation of multiple signaling pathways, such as the RAS/MAPK, PI3K/AKT, and protein kinase C (PKC) signaling pathways [154,164]. 

The PKC pathway is involved in controlling numerous cellular functions such as cell growth, differentiation, survival, and apoptosis. The PKC family consists of at least ten isoforms that are categorized into three groups: classical (α, βI, βII, and γ), novel (δ, ε, η, and θ), and atypical (ζ and ι/λ). The deregulation of PKC signaling has been associated with the onset and advancement of various types of cancer, including breast, lung, colon, and pancreatic cancer. PKC isoforms have been found to play a role in cancer cell proliferation, invasion, metastasis, and resistance to chemotherapy and radiation therapy [165]. Multiple types of research have indicated that the PKC pathway plays a crucial role in cancer development, and different treatment methods aimed at PKC signaling are currently being researched. One of these approaches involves the application of PKC inhibitors, which have exhibited encouraging outcomes in both preclinical studies and clinical trials, implying that targeting the PKC pathway could be a viable cancer therapy method [166].

It was shown that EGCG may work as a tyrosine kinase inhibitor. For instance, it has been observed that advanced non-small-cell lung cancer (NSCLC) showed a good response to EGCG treatment. EGCG not only decreased the EGFR expression but also its downstream protein phosphorylation. However, those results were observed in a cell line with a wild-type *EGFR* gene [167]. It appears that mutations occurring in *EGFR*, which are common in NSCLC, influence EGCG treatment efficacy. In a study that compared the sensitivity of three different NSCLC cell lines to EGCG, resistance to treatment was observed for two cell lines harboring mutations in *EGFR*, indicating a strong association between the anticancer activity of EGCG and *EGFR* mutation status. Mutations in *EGFR* appeared to have an impact on its ability to bind ECGC, so the receptor still could undergo phosphorylation [11]. Nevertheless, the results remain inconclusive, as there are also studies reporting no significant difference in the mutated *EGFR* cell line’s response to ECGC [168].

As described in further investigations, EGCG was tested in various types of cancer cells, usually with the outcome indicating that EGCG blocks the EGFR pathway via binding with the EGFR receptor. In a study examining its impact on three human thyroid carcinoma cell lines—TT, TPC-1, and ARO—EGCG was shown to decrease the protein levels of phosphorylated EGFR in concentrations of 50–200 µM [169]. In hepatocellular carcinoma cells, a down-regulation of EGFR expression and one of its ligands—HER2—was observed following treatment with EGCG. However, no clear dose dependency was established [170]. Moreover, a short pretreatment (30 min) with 100 and 200 µM EGCG was proven to partially inhibit the EGFR phosphorylation induced by EGF in an A-431 epidermoid carcinoma cell line [171]. Furthermore, in a human salivary gland adenoid cystic carcinoma cell line SACC-38, EGCG (in concentrations of 5, 10, 20, 40, and 80 μM for 48 h) significantly decreased EGFR protein levels and inhibited EGFR phosphorylation [172].

#### 2.4.2. JAK/STAT Pathway

The JAK/STAT signaling pathway constitutes one of the major cell signaling hubs in cellular homeostasis. This evolutionarily conserved pathway can be activated by numerous hormones, growth factors, interferons (IFNs), interleukins (ILs), and colony-stimulating factors and is involved in the control of a variety of processes, including apoptosis, survival, and inflammation [173,174]. In the traditional JAK/STAT pathway, the interaction between the ligand and its receptor triggers the dimerization and transphosphorylation of the second. This facilitates the formation of a docking site for STATs. JAK phosphorylates STATs at this docking location, and after that, STAT dissociates from the receptor and forms homodimers or heterodimers through interactions involving the SH2 domain and phosphotyrosine. These dimers then translocate to the promoters of the target genes, where they regulate their transcription. STATs either bind to their DNA target sites to activate transcription or associate with non-STAT DNA-binding factors to promote STAT-dependent transcription. Both STAT and non-STAT transcription factors can synergistically activate transcription by binding to clusters of independent DNA-binding sites. The deregulation of this pathway has been frequently linked to various malignancies including breast cancer, glioblastoma, hepatocellular carcinoma, Hodgkin’s lymphoma, myeloproliferative neoplasm, natural killer/T-cell lymphoma, non-small-cell lung cancer, prostate cancer, thyroid cancer, and others, as previously reviewed by multiple authors [175,176,177,178,179].

According to research carried out by Tang and colleagues, EGCG inhibited the expansion, invasion, and migration of pancreatic cancer cells while simultaneously inducing apoptosis via interfering with the STAT3 signaling pathway in vitro. In addition, EGCG contributed to an increase in the gemcitabine and CP690550 efficacy in AsPC-1 and PANC-1 pancreatic cancer cell lines [180]. It was also found that the treatment of breast cancer and head and neck cancer cell lines with EGCG decreased the phosphorylation of the ERK, STAT3, and EGFR proteins. The fact that EGCG induced a decrease in the level of BCL-2 and BCL-X(L) proteins and increased BAX protein expression and the activation of caspase 9 lends credence to the hypothesis that EGCG triggers apoptosis by the mitochondrial apoptosis pathway. The growth-inhibiting effects of 5-fluorouracil were significantly amplified by the addition of EGCG. These findings provide insights into the molecular mechanisms of growth suppression by EGCG and suggest that this molecule may be effective in the chemoprevention and/or therapy of breast cancer and head and neck squamous cell carcinoma (HNSCC) when used alone or in conjunction with other drugs [13,181]. It has been discovered that EGCG can limit the proliferation of chronic myeloid leukemia cells by inducing apoptosis via the suppression of the BCR/ABL oncoprotein and managing its downstream pathways, such as p38-MAPK/JNK and JAK2/STAT3/AKT [182]. The pretreatment of cholangiocarcinoma (CCA) cells with quercetin and EGCG inhibited the activation of the JAK/STAT pathway caused by the IL-6 and INF-γ treatment. This was demonstrated by a dose-dependent reduction in the levels of phosphorylated STAT1 and STAT3 proteins. The quercetin and EGCG pretreatments were successful in inhibiting the cytokine-mediated up-regulation of iNOS and intercellular adhesion molecule-1 (ICAM-1) via the JAK/STAT cascade. In addition, these flavonoids have the potential to suppress the proliferation of CCA cells as well as cytokine-induced cell migration [183]. Experiments conducted in in vitro settings have shown that treatment with EGCG suppressed INFγ-induced JAK-STAT signaling as well as the production of immune-checkpoint molecules, namely programmed death-ligand 1 and 2 (PD-L1 and PD-L2). It was demonstrated that this impact was caused by a decreased expression of STAT1 as well as its phosphorylation, which led to a reduction in the activity of the regulator interferon regulatory factor 1 (IRF1) for PD-L1/PD-L2 in human and mouse melanoma cells. The in vivo tumor-inhibitory action of EGCG was shown to be mediated by CD8+ T lymphocytes, and the effect was comparable to that of anti-PD-1 therapy in animals. However, their modes of action were rather distinct from one another. EGCG was able to decrease JAK/STAT signaling as well as PD-L1 expression in tumor cells, which resulted in the reactivation of T cells. This was in contrast to the anti-PD-1 treatment, which prevented the interaction between PD-1/PD-L1. In conclusion, it was shown that EGCG inhibits JAK-STAT signaling in melanoma, which in turn increases anti-tumor immune responses [15]. By using surface-plasmon-resonance (SPR)-binding assays and in silico docking studies, Wang et al. showed that EGCG disrupted STAT3 peptide binding at micromolar concentrations, and the docking experiments showed that EGCG interacts with Arg-609, which is one of the pivotal residues in the STAT3 SH2 domain responsible for STAT3 and phosphorylated peptide binding [184]. A significant inhibition of tyrosine and serine phosphorylation of STAT 1 and STAT3 was seen in human oral cancer cells [185,186] and cholangiocarcinoma cells [183] treated with EGCG.

#### 2.4.3. MAPKs Pathways

MAPK pathways influence gene expression and play an essential role in plenty of cellular processes that decide a cell’s fate, such as proliferation, differentiation, apoptosis, survival, and other stress responses. MAPK pathways consist of a three-step kinase cascade of MAP3K, MAP2K, and MAPK kinases and are tightly regulated by other factors including phosphatases as well as their pseudoenzyme equivalents [187,188]. Traditionally, among the most recognized MAPK pathways, ERK is classified as a mitogen-responsive MAPK, while JNK and p38 are MAPKs engaged in a reaction to stressors. However, in reality, the response pathways often overlap. In total, 40% of all human cancers have RAS-RAF-MEK-ERK pathway malfunctions, which makes MAPK pathways an excellent target of anti-cancer therapies [189].

EGCG was found to inhibit the p38-MAPK/JNK pathway in chronic myeloid leukemia cells in a dose-dependent manner. The experiment was carried out on five cell lines—K562, K562R, KCL-22, BaF3/p210, and BaF3/p210T315I—including those unresponsive towards a currently used chemotherapeutic, imatinib. Not only was EGCG effective in imatinib-resistant cells, causing a decrease in the cells’ viability, but it was also more cytotoxic towards leukemic cells in comparison to bone marrow mononuclear cells isolated from healthy donors. Interestingly, it was observed that EGCG-induced apoptosis was triggered by the apoptosis-inducing factor (AIF) and was executed in a caspase-independent pathway, including autophagy. EGCG was shown to down-regulate both P38 and phosphorylated P38 levels in leukemic cells while up-regulating phosphorylated JNK, though did not influence JNK and ERK protein levels [182].

Though EGCG is usually believed to inhibit MAPK pathways, in an experiment on hepatocellular carcinoma cells, it caused the phosphorylation of ERK. It was shown that a prolonged activation of ERK induced cell apoptosis [170]. Moreover, when EGCG was applied to a hepatocarcinoma cell line (HepG2), its proapoptotic activity was stimulated by the JNK pathway [190]. However, in a study on human bladder cancer cells (T24), a 30 min pretreatment with EGCG before the application of IL-1β caused the suppression of both the ERK1/2 and JNK pathways by silencing their transcriptional activity, while EGCG, in a concentration range of 0–50 μM, did not affect the T24 cells’ viability [191]. In a study by Chen et al., EGCG was indicated as an estrogen receptor (ERα36) inhibitor in Hep3B cells, where it showed both anti-proliferative and pro-apoptotic properties, leading to the inhibition of the ERα36-EGFR-HER-2 feedback loop or the PI3K/AKT and MAPK/ERK pathways [170].

#### 2.4.4. PI3K/AKT/mTOR Pathway

The PI3K/AKT/mTOR signaling hub regulates a wide variety of cellular activities, including cell survival, proliferation, growth, metabolism, angiogenesis, and metastasis. This pathway is hyperactivated or disrupted in many kinds of cancer [192]. Signaling from receptor tyrosine kinases or the binding of growth factors to PI3Ks trigger the phosphorylation of the hydroxyl groups (3′OH) of phosphatidylinositol. Phosphorylation of phosphatidyl-1,4-bisphosphate (PIP2) leads to the formation of phosphatidylinositol-1,4,5-triphosphate (PIP3), which recruits phosphoinositol-dependent kinase 1 (PDK1) and AKT kinase to the vicinity of the cell membrane. The phosphorylated, active AKT phosphorylates proteins involved in cell growth and survival and induces the expression of many proteins with proliferative and anti-apoptotic functions, including BCL-2, BCL-xL, and NF-Kb. Another growth regulator activated downstream of PI3K/AKT is mTOR serine/threonine kinase. AKT activates mTOR, which then phosphorylates ribosomal protein kinase S6 (S6K) and inactivates eukaryotic translation initiation factor 4E binding protein (4E-BP1). mTOR coordinates cell growth mainly through increased mRNA translation. An important regulator of the pathway is the phosphatidylinositol 3,4,5-trisphosphate 3-phosphatase and dual-specificity protein phosphatase (PTEN) protein, which dephosphorylates PIP3 and thus prevents the activation of the AKT protein. The details of the PI3K-AKT-mTOR signaling network and its targeting were previously and extensively described by other authors [193,194,195,196,197,198] and will not be discussed here in detail.

**Figure 4:**
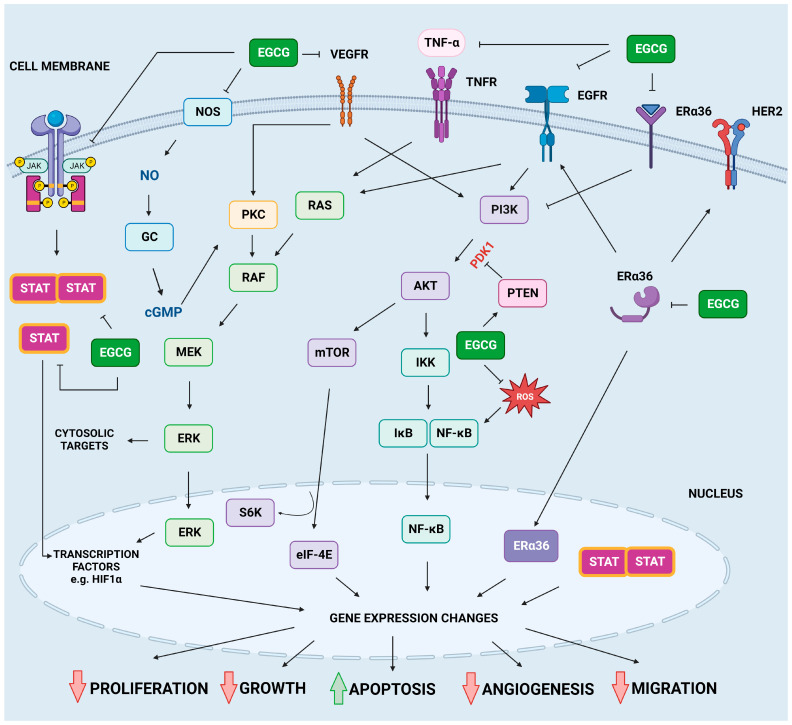
Overview of inhibitory effects of EGCG on key signaling pathways associated with the development of cancer. The details can be found in the main text of the manuscript. For the full names of the proteins, see the abbreviations section. Created with BioRender.com, accessed on 29 March 2023.

EGCG can inhibit the proliferation of pancreatic carcinoma PANC-1 cells and bladder cancer T24 and 5637 cells or promote their apoptosis through the up-regulation of PTEN expression while simultaneously suppressing the phosphorylation and expression of AKT and mTOR [38,199]. Similar observations were made when the effect of EGCG was investigated in lung cancer cells (H1299 cell line), where a down-regulation of phosphorylated PI3K and AKT was shown following EGCG treatment [22]. The study of Ding and Yang also examined whether EGCG inhibits colon cancer cell growth in vitro and nude mouse xenografts by targeting the sonic hedgehog (SHH) and PI3K pathways. EGCG inhibited colon cancer cell proliferation dose-dependently with negligible toxicity to normal colon epithelial cells. EGCG suppressed colon cancer cell migration and invasion and induced their apoptosis. Furthermore, the use of purmorphamine (smoothened (Smo) agonist) or IGF-1 (PI3K agonist) partially blocked EGCG’s inhibitory effects on cell proliferation, migration, and death [200]. Numerous other studies have indicated that EGCG induces apoptosis via the inhibition of the PI3K/AKT/mTOR pathway, which leads to the promotion of cytotoxic autophagy. The anti-cancer properties of EGCG have also been hypothesized to be caused by its pro-oxidant activity. When used in conjunction with chemotherapy treatment, EGCG suppressed both the proliferation of cancer cells and the drug’s induced pro-survival autophagy, as recently reviewed by Ferrari et al. [201]. An overview of the inhibitory effects of EGCG on the key signaling pathways associated with the development of cancer is shown in Figure 4.

#### 2.4.5. Other Signaling Pathways

The 67-kDa laminin receptor (67-LR) is located on the cell surface and is a key factor in cell adhesion tumor growth, invasion, and metastasis [202,203]. Its overexpression is found in various types of tumors, and recently 67-LR has been appointed as a single-target molecule interacting with EGCG at physiologically achievable concentrations (0.1–1 μM) [204,205]. Hepatoma cells treated with radiotherapy can develop invasive potential due to the activation of hepatic stellate cells. It was observed that a 48 h EGCG pretreatment of LX-2 cells (human hepatic stellate cell line) inhibited the toll-like receptor 4 (TLR4) pathway by binding with 67-LR. This resulted in diminished invasive properties of the irradiated cells [206]. Lipid rafts are microdomains of the cell membrane that are rich in cholesterol and sphingolipids and play an important role in cell signaling and communication. Cellular functions such as adhesion, migration, and proliferation have all been linked to these microdomains. Lipid rafts have been linked to tumor development, invasion, and metastasis. Lipid raft disruption can alter the positioning and function of signaling molecules like EGFR and HER2 that play a role in cancer cell proliferation and survival. Through enhancing invadopodia formation and metalloproteinase activity, lipid rafts can also encourage cancer cell movement and invasion. It has been suggested that lipid rafts could be used as a target in the treatment of cancer [207,208,209,210]. Treating human multiple myeloma U266 cells with EGCG resulted in plasma membrane lipid raft disruption via the 67-LR/acid sphingomyelinase (ASM) pathway and the elicitation of EGCG-induced cell death. Moreover, the pretreatment of cells with anti-67-LR antibodies protected them from EGCG influence. As 67-LR is overexpressed on the surface of cancer cells, EGCG’s cell-killing properties appear to be cancer cell-specific. However, high doses of EGCG are hepatotoxic, and the concentration of EGCG needed for activating the lipid raft disruption process seems unachievable in therapy. Sphingosine kinase-1 inhibitor (safingol) significantly sensitizes multiple myeloma cells to EGCG and could help in reducing the dose of the phytochemical [211]. EGCG triggered the protein phosphatase 2A (PP2A) pathway via 76-LR interaction in melanoma cells (Hs294T, A375, MeWo cell lines), thus limiting tumor growth. As in earlier studies, primary normal human melanocyte cells were unaffected, confirming EGCG’s cancer cell discrimination [212].

Energy homeostasis in cells is controlled by the AMP-activated protein kinase (AMPK) pathway. The activation of AMP-activated protein kinase (AMPK) suppresses cell growth and proliferation by inhibiting anabolic pathways and stimulating catabolic pathways. The dysregulation of the AMPK pathway has been linked to cancer progression, metastasis, and drug resistance. The activation of AMPK has been proven in multiple studies to reduce the survival and growth of cancer cells. Important steps in the development of cancer, such as angiogenesis and inflammation, can be suppressed by activating AMPK. In addition, activating AMPK can make cancer cells more sensitive to chemotherapy and radiotherapy, making it a promising target for cancer treatment. However, other research has revealed that AMPK activation can increase tumor development and survival in specific circumstances, suggesting that AMPK’s role in cancer may be nuanced and situation-specific [213,214,215]. There is mounting evidence that EGCG can stimulate ROS production, which in turn leads to the phosphorylation and activation of AMPK. Some of the proteins that are involved in adipogenesis, lipogenesis, and lipolysis can be modulated by phosphorylated AMPK in response to EGCG treatment. This is known as the “AMPK hypothesis”, which was proposed by Yang et al. [216]. AMPK activation by EGCG can lead to the inhibition of the mTOR pathway, which is involved in regulating cell growth and proliferation, as well as the activation of autophagy, which can promote cell death. EGCG-mediated activation of AMPK has also been shown to sensitize cancer cells to chemotherapy and radiotherapy [217,218].

Focal adhesion kinase (FAK) is a protein that plays a critical role in cell signaling and the regulation of cell behavior. The FAK pathway is involved in several cellular processes including cell adhesion, migration, proliferation, and survival. The dysregulation of FAK signaling has been linked to the development and progression of several types of cancer including breast, lung, and pancreatic cancer. FAK is known to promote tumor growth, invasion, and metastasis as well as resistance to chemotherapy and radiotherapy. Targeting the FAK pathway has emerged as a promising approach for cancer therapy [219,220,221]. EGCG treatment can suppress FAK activity, leading to reduced cancer cell migration, invasion, and proliferation. Moreover, EGCG-mediated inhibition of the FAK pathway has been demonstrated to sensitize cancer cells to chemotherapy and radiotherapy. These findings suggest that EGCG has potential as an anti-cancer agent that targets FAK signaling in cancer cells [222,223,224].

The hedgehog (Hh) signaling pathway plays a crucial role in cell growth, differentiation, and tissue development. The aberrant activation of this pathway has been linked to various types of cancer, including basal cell carcinoma, medulloblastoma, and gastrointestinal, lung, breast, and prostate cancers. Activation of the Hh pathway in cancer leads to an increased expression of target genes that promote cell proliferation, survival, and invasion. Thus, targeting the Hh pathway has emerged as a promising strategy for cancer therapy [225,226]. The expression of Hh pathway components such as smoothened (SMO) and GLI1 have been discovered to be suppressed by EGCG, ultimately leading to the inhibition of the Hh pathway [227]. EGCG has been proven to reduce the proliferation, migration, and invasion of cancer cells through the blockade of the Hh pathway [41,200].

The NOTCH pathway is a signaling pathway that is important in determining cell fate, proliferation, and differentiation. Its malfunction has been implicated in the growth and spread of different types of cancer including leukemia, lung cancer, and breast cancer. The key components of the NOTCH pathway include NOTCH receptors (NOTCH1-4), ligands (JAGGED1, JAGGED2, DELTA-like1, DELTA-like3, and DELTA-like4), and downstream transcription factors such as HES and HEY. The abnormal activation of the NOTCH pathway has been linked to increased cancer cell growth, survival, invasion, and resistance to chemotherapy and radiation therapy. On the other hand, blocking the NOTCH pathway has been shown to trigger cancer cell differentiation and cell death, and it makes them more responsive to therapy. Hence, inhibiting the NOTCH pathway has become a promising approach for treating cancer, and various NOTCH pathway inhibitors are currently under development for cancer treatment [228,229,230]. EGCG was found to be a NOTCH signaling inhibitor [231]. For example, Wei et al. found that by preventing the activation of the NOTCH signaling system, EGCG dramatically reduced the proliferation of and promoted apoptosis in tongue cancer cells [232]. In another study, EGCG was shown to influence the cell cycle, trigger apoptosis, and suppress the growth of colorectal cancer cells in vitro and in vivo. The expressions of HES1 and NOTCH2 were suppressed after EGCG treatment [65].

Mutations in the genes encoding components of the WNT/β-catenin pathway, such as APC and β-catenin, can cause the accumulation of β-catenin in cancer cells. This accumulation results in the activation of the WNT/β-catenin pathway, which encourages cell proliferation, survival, and invasion. This pathway’s dysfunction has been linked to various cancers, including colon, liver, breast, and lung cancers [233,234]. EGCG was found to exercise its anticancer action by boosting the phosphorylation and proteasomal degradation of β-catenin through a mechanism that is independent of both glycogen synthase kinase-3 (GSK-3) and protein phosphatase 2A (PP2A). Zhu et al. found that EGCG down-regulated WNT/β-catenin activation in lung cancer stem cells [235].

## 3. Emerging EGCG Targets

In the early phases of the drug design process, computational techniques supplement experimental analysis by providing important insights into the comprehension of the molecular mechanisms of compound activity. Molecular docking is a computational strategy used to anticipate the binding modes of small compounds or macromolecules with a receptor. This methodology allows the ranking of compounds based on a hierarchy established by specific scoring functions. When coupled with the more expensive but also more precise molecular dynamics (MD) techniques, docking gains a major complementary tool [236]. This approach has been found to be useful in the identification and optimization of many currently approved drugs including captopril, oseltamivir, and orzanamivir [237].

Glucose-regulated protein 78 (GRP78) is a crucial component of the unfolded protein response. It works as a chaperone protein and is up-regulated in cancer cells, conferring the inhibition of apoptosis and the promotion of chemoresistance in treated cancer cells. GRP78 is composed of an ATPase domain, a substrate-binding domain, and a linker region. ATP-competitive inhibitors such as EGCG suppress GRP78 activity and hinder its expression in glioblastomas. The in silico studies of Bhattacharjee et al. revealed crucial amino acids involved in GRP78-EGCG binding, including Ile61, Glu293, Arg297, and Arg367 [238]. Furthermore, apoptosis induction, MMP changes, and ROS reduction were observed in colorectal cancer cells treated with EGCG alone or in conjunction with irinotecan. When given alongside irinotecan, EGCG has been shown to increase the chemosensitivity of colon cancer cells by inducing GRP78-mediated endoplasmic reticulum stress [63]. Similar observations were made in breast cancer cells treated with microtubule-interfering agents (taxol and vinblastine), further confirming the role of EGCG as a GRP78 inhibitor [239,240].

Khan et al. used a structure-based virtual screening of phytocompounds to identify possible inhibitors of hexokinase 2 (HK2) [241]. HK2 is the most active of the family’s isozymes and is primarily expressed in insulin-sensitive tissues. The induction of HK2 in the majority of neoplastic cells leads to their metabolic shift towards aerobic glycolysis, and the genetic deletion of HK2 prevented the progression of malignant growth in mouse models [242]. EGCG and quercitin were identified as two compounds with a significant capability of HK2 inhibition in docking and MD simulations. Based on the outcomes of MD simulations, it was discovered that HK2 consistently creates stable protein–ligand complexes with EGCG and quercitrin throughout the simulation process. In general, these findings point to the possibility that EGCG and quercitrin could be further investigated as potential scaffolds in the process of developing drugs that target HK2 [241]. This is consistent with earlier studies on the impact of EGCG on glycolysis, where the treatment of human tongue carcinoma cells resulted in the down-regulation of HK2 [243]. Other studies suggested a more versatile function of EGCG in glycolysis, where it reduced the activity and expression of several enzymes including phosphofructokinase (PFK) and pyruvate kinase (PK) in pancreatic cells in vitro; however, this was without the influence of HK expression. Furthermore, EGCG treatment resulted in a decrease in the levels of platelet-type phosphofructokinase (PFKP) and pyruvate kinase M2 (PKM2) in pancreatic tumor xenograft homogenates acquired from mice [244].

In chronic lymphocytic leukemia (CLL), the expression of zeta-chain-associated protein kinase-70 (ZAP-70) is a significant component in the development of a poor prognosis of the disease. This protein tyrosine kinase is an important mediator in the signaling process that is mediated by the T-cell receptor (TCR). Structurally, it is homologous to spleen tyrosine kinase (SYK), which plays a similar function in the signaling process that is mediated by the B-cell receptor (BCR) [245]. Recent evidence also indicates that ZAP-70, which is intrinsically expressed in tumor cells, may influence the crosstalk between malignant B cells and the immune microenvironment. This suggests that ZAP-70 plays a more complicated role in the pathogenesis of B cell malignancies than was previously thought [246]. Additional data showed that EGCG effectively suppressed ZAP-70 activity in CD3-activated T-cell leukemia, demonstrating a high binding affinity (Kd = 0.6207 μM). Furthermore, EGCG therapy dose-dependently suppressed the CD3-induced activation of activator protein-1 (AP-1) and IL-2. In particular, EGCG triggered caspase-mediated apoptosis in ZAP-70-expressing leukemia cells in a dose-dependent manner, whereas EGCG treatment was ineffective against P116 ZAP-70-deficient cells. The stability of the ZAP-70-EGCG complex may be aided by a network of intermolecular hydrogen bonds and hydrophobic contacts, as demonstrated by site-directed mutagenesis and molecular docking studies [247].

Wang et al. [248] employed a reverse docking methodology to identify new targets of EGCG and comprehend the pharmacological mechanism of action of this compound. The research revealed that EGCG may impact a total of 12 signaling pathways and 33 proteins. EGCG was found to exert binding properties with many proteins previously investigated in in vitro studies including proto-oncogene tyrosine-protein kinase FYN (FYN) [249], NOS2 [250], cyclin-dependent kinase 2 (CDK2) [251,252], tyrosine-protein kinase ABL1 (ABL1) [253], SYK [254], AKT1/2 [18,255], MAPK8, [256], interleukin-1 receptor-associated kinase 4 (IRAK4) [257], and apoptotic protease-activating factor 1 (APAF1) [258]. Furthermore, using network pharmacology, molecular dynamics simulation, and enzymatic assays, authors have investigated the inhibition of novel potential EGCG targets that were previously not investigated, including IKKβ, GTPase KRas (KRAS), high-affinity nerve growth factor receptor (NTRK1), and Wee1-like protein kinase (WEE1). EGCG has been shown to moderately suppress WEE1 activity while efficiently inhibiting IKKβ, KRAS, and NTRK1 in enzymatic assays [248]. The WEE1 kinase is essential for DNA repair at the G2-M cell-cycle checkpoint and is overexpressed in a wide range of malignancies, including, but not limited to, breast cancer, leukemias, melanomas, adult, and brain tumors [259]. In contrast, mutations in KRAS result in the permanent activation of this protein that acts as a molecular switch to consistently stimulate downstream signaling pathways controlling cell proliferation and survival [260,261]. On the other hand, NTRK1 is involved in the control of neural development and differentiation. In multiple tumor types, a constitutive activation of NTRK1 was observed. Tumor progression in both prostate carcinoma and breast cancer has been linked to an autocrine cycle involving NTRK1. Recently, a novel alternative splicing variant in neuroblastoma was characterized. Moreover, a significant proportion of papillary thyroid cancers harbor NTRK1 somatic rearrangements that generate chimeric oncogenes with constitutive tyrosine kinase activity, as reviewed by Pierotti and Greco [262]. Several other computational studies have investigated the effects of EGCG on other cancer-related factors including trypsin [263], proteasome [264], PTP1B phosphatase [265], glutathione S-transferase (GST P1-1) [266], or the epigenetic modulators DNMT [267], HDAC [57], and sirtuin-6 (SIRT6) [268], as recently reviewed [269,270].

## 4. EGCG in Cancer Prevention and Therapy?

### 4.1. Epidemiological Studies

The results of epidemiological studies on the relationship between drinking tea and the risk of developing cancer in human subjects have been inconclusive. According to Yuan et al., drinking black tea did not seem to reduce the incidence of cancer. After accounting for any confounding factors, a high consumption of green tea was consistently related to a lower risk of malignancies of the upper gastrointestinal tract and could help to prevent lung and liver cancer. No conclusive data suggest that tea consumption helps to prevent the onset of malignancies of the colorectum, pancreas, and urinary system or the development of glioma, lymphoma, or leukemia [271]. According to Zhang et al., consuming additional quantities of tea did not significantly reduce the chance of developing gliomas, brain tumors, stomach cancer, colon cancer, lung cancer, pancreatic cancer, liver cancer, breast cancer, prostate cancer, ovarian cancer, or bladder cancer. On the other hand, heavy tea consumption was connected to a decreased incidence of oral cancer. In addition, in western countries, an increase in tea consumption was associated with a lower bladder cancer risk [272]. While some studies in Asian populations indicated positive associations between green tea consumption and mortality [273], unclear results were obtained in epidemiological studies investigating the impact of green tea consumption on colorectal cancer [274], esophageal cancer [275,276], and gastric cancer [277] incidence in other populations.

### 4.2. Clinical Studies

The clinical trial results obtained in the last ten years of research indicate the beneficial influence of EGCG on the human body. Studies have focused on the influence of this phenolic compound on amyloidosis [278] atherosclerosis [279], cystic fibrosis [280,281], Barrett’s oesophagus [282], diabetes [283,284,285], Down’s syndrome [286,287,288,289], dystrophic epidermolysis bullosa [290], hypertension [291,292], multiple sclerosis [293,294,295,296,297,298,299,300], obesity [301,302,303,304,305,306,307,308], radiation-induced esophagitis [309,310,311,312], and ulcerative colitis [313].

Importantly, green tea extracts and tea consumption cannot be considered equal for several reasons. Green tea extracts are highly concentrated forms of the active compounds found in green tea, whereas tea consumption involves drinking brewed green tea, which typically contains lower concentrations of these compounds. Extracts are often made by concentrating the beneficial components of green tea, such as catechins, into a supplement form. Green tea extracts can be standardized to contain specific amounts of active compounds, ensuring consistency in dosage and potency. On the other hand, the composition of brewed tea can vary depending on factors such as brewing time, temperature, and the specific tea leaves used, making it challenging to determine the exact concentration of active compounds. Similarly, the extraction conditions may strongly impact the content of bioactive compounds in the final product. Furthermore, when drinking brewed green tea, the consumption of other components present in the tea leaves occurs. These include caffeine and various antioxidants, which can have additional effects on the body. Green tea extracts, on the other hand, may be formulated to remove or reduce certain components, allowing for the targeted supplementation of specific compounds [314,315,316,317].

Liu et al. investigated the effect of an intravesical irrigation of EGCG (dosage: 4 mg/kg body weight once per week with four treatments in total) in patients diagnosed with interstitial cystitis. The anticancer activities of this chemical against bladder cancer were attributed to the molecule’s ability to block the expression of purinergic receptors in urothelial cells as well as the release of ATP from those cells. In addition, EGCG inhibited the induction of iNOS and phosphorylated NF-κB, which resulted in the significant antioxidative and anti-inflammatory activity of the compounds [318]. An EGCG-enriched tea drink—double-brewed green tea (DBGT)—was the subject of a phase II study that was designed to evaluate the efficacy and safety of the beverage as a maintenance treatment for women who had advanced-stage serous or endometrioid ovarian cancer. After standard treatment for ovarian cancer, DBGT supplementation did not appear to be a promising maintenance option for patients who had reached an advanced stage of the disease [319].

Some data from preclinical studies, epidemiologic studies, and previous clinical trials suggest that catechins found in green tea may lessen the chance of developing prostate cancer. Although being well tolerated and accumulating in plasma over a year, a standardized, decaffeinated catechin mixture comprising 400 mg of EGCG per day did not diminish the risk of prostate cancer in men who had high-grade prostatic intraepithelial neoplasia (HGPIN) and/or atypical small acinar proliferation (ASAP) at the start of the study [320]. However, ASAP was never identified as a pre-neoplastic lesion in the context of prostate cancer [321,322]. When the same data from Kumar et al. [320] was analyzed without the ASAP lesions but with the HGPIN lesions, the reduction in PCa progression was approximately 50% according to a Kaplan-Meyer analysis. Furthermore, Bettuzzi et al. [323]. found a 90% reduction in prostate cancer in men with HGPIN who received green tea catechins for a year. Furthermore, the authors found no toxicity with green tea catechins in men with HGPIN who were randomly assigned to receive 600 mg of EGCG per day for a year. This chemoprevention strategy in high-risk patients would address a huge therapeutic and societal need, paving the way for a unique and effective clinical approach [323,324,325]. This is consistent with the findings of Parletti et al. [326], who conducted a single-outcome meta-analysis on the preventive role of green tea catechin extracts towards prostate cancer. The total number of patients in the study was 223; 114 and 109 were assigned to the catechin and placebo groups, respectively. There were nine incidences of prostate cancer in the catechin arm (7.9%) and twenty-four cases in the placebo arm (22%). Pooled analysis revealed that the catechin arm had a significantly lower cancer risk (risk-ratio = 0.41; 95% CI: 0.19–0.86; I^2^ = 0). These findings indicate that consuming concentrated green tea catechin formulations may provide significant protection to carriers of early neoplastic lesions of the prostate. The evidence is of average quality, and further large-scale research is needed to back up these preliminary conclusions [326].

Another study indicated that tea polyphenols and theaflavins (including EGCG) are bioavailable in the prostates of cancer patients, where they may play a role in prostate cancer prevention. For 5 days before radical prostatectomy, 20 men were randomized to consume 1.42 L of green tea, black tea, or a caffeine-matched soda control daily. Tea polyphenol levels were higher in the prostate samples from the individuals who consumed black tea and green tea versus the men who consumed the soda control. While tea polyphenols were not detected in serum, ex vivo LNCaP prostate cancer cell proliferation was reduced when cells were cultured in conditions containing patient sera collected after black tea and green tea consumption compared to baseline serum [327]. Additionally, men with clinically localized prostate cancer were given six cups of green tea or water daily for three to six weeks before undergoing radical prostatectomy to explore the metabolism and bioactivity of green tea polyphenols in human prostate tissue. Following a short-term green tea treatment, methylated and nonmethylated forms of EGCG were detected in prostate tissue, and the degree of methylation of EGCG might modulate its preventive effect on prostate cancer, probably involving genetic polymorphisms of catechol O-methyltransferase [328]. McLarty et al. investigated the short-term supplementation of polyphenon E (PPE) comprising 800 mg of (−)-epigallocatechin-3-gallate (EGCG) on the protein levels of hepatocyte growth factor (HGF), VEGF, IGF-I, IGF binding protein-3 (IGFBP-3), and prostate-specific antigen (PSA) in prostate cancer patients’ sera. The results demonstrated a significant reduction in PSA, HGF, and VEGF serum levels in males with prostate cancer after a short course of treatment with EGCG (PPE) with no increase in liver enzymes. These data suggest the possible role of PPE in the therapy or prevention of prostate cancer [329].

Fatty acid synthase (FAS) is essential for the development and maintenance of tumors with lipogenic characteristics because of its role as a master regulator of lipid metabolism. Evidence suggests that it can rewire tumor cells to have more energy flexibility, which is necessary for them to meet their high energy needs [330]. In contrast, the Ki-67 protein has served as a standard proliferation marker for human tumor cells for many years. Multiple molecular roles of this immense protein have been elucidated in recent research [331]. According to the findings of another clinical trial, supplementation with fish oil (1.9 g pf docosahexaenoic acid (DHA) + eicosapentanoic acid (EPA)/day) or EGCG (600 mg/day) for 90 days may not be adequate to generate biologically significant changes in FAS or Ki-67 protein levels in prostate tissue [332]. It was also found that a daily intake of a predefined, decaffeinated catechin mixture containing 200 mg of EGCG taken with food for one year produced an accumulation of the phenolic compound in plasma, and it was well tolerated and did not produce any treatment-related adverse effects in men who had baseline HGPIN or ASAP [325]. The above-mentioned studies investigated the effects of relatively short supplementation. In another study in men with baseline HGPIN or ASAP, daily intake of a uniform, decaffeinated catechin PPE composition comprising 200 mg/twice a day, EGCG that was taken with food for 1 year accumulated in plasma, was well tolerated, and did not elicit treatment-related side effects [325].

Also, the administration of GTE capsules (800 mg EGCG) seems to regulate biomarkers related to colorectal cancer pathogenesis, especially genes associated with selenoproteins, WNT signaling (β-catenin), inflammation (NF-κB), and methylation (DNMT1) [333]. Based on the synergistic effects seen in preclinical studies between green tea PPE and EGFR-tyrosine kinase inhibitors [334], a 6-month phase IB study of PPE (200 mg three times a day) and erlotinib (dose escalation of erlotinib (50, 75, and 100 mg daily)) was conducted in patients with head and neck cancer who had advanced premalignant lesions (APL) of the oral cavity and larynx. Patients diagnosed with APLs of the head and neck who received treatment with the combination of PPE and erlotinib were reported to have a prolonged cancer-free survival. The regimen was well tolerated by the patients, indicating that this combination should be investigated further for its potential to be used as a chemoprevention of recurrent primary tumors in early-stage head and neck cancer patients [335]. Several other clinical studies are planned to investigate EGCG’s anticancer activity and safety in cancer patients, as discussed in Table 1.

### 4.3. Bioavailability of EGCG

One of the obstacles to the clinical use of EGCG is its poor oral bioavailability that is related to its low intestinal absorption and short retention time or lack of stability. Overcoming these challenges related to its physicochemical properties will realistically impact the conversion of EGCG from basic studies to widespread use in clinical settings [336]. Improvements in EGCG’s pharmacokinetics and pharmacological properties, and thus its therapeutic possibilities, have been achieved by a variety of approaches, including structural alterations of the compound, the use of nano-carriers including carbohydrate-based carriers (chitosan-tripolyphosphate nanoparticles [337], γ-cyclodextrin (γ-CD), or carriers coated with hydroxypropylmethyl cellulose phthalate [338,339]), lipid carriers (liposomes) [340,341,342], protein carriers [343,344,345,346], and other effective drug delivery methods, as recently discussed in excellent reviews [347,348]. These approaches increase the bioavailability of EGCG and prolong the dosing interval [349].

EGCG has been investigated in doses ranging from 150 to 400 mg per day, with oral administration being the most extensively utilized delivery modality in animal models as well as clinical studies [350,351,352]. In contrast, it was reported that doses of 140 mg to 1000 mg per day may exhibit hepatotoxic effects [353]. However, these finding seems ambiguous [354]. Lambert et al. investigated the hepatotoxicity of large doses of EGCG in male CF-1 mice. A single dose of EGCG (1500 mg/kg, i.g.) elevated plasma ALT levels by 138-fold and lowered mice survival by 85%. Once-daily EGCG administration increased the level of hepatotoxic reaction. Following two once-daily dosages of 750 mg/kg EGCG, plasma ALT levels were elevated 184-fold. Following EGCG therapy, moderate-to-severe hepatic necrosis was found; elevated hepatic lipid peroxidation (5-fold increase), plasma 8-isoprostane (9.5-fold increase), and elevated hepatic metallothionein and gamma-histone 2AX protein expression were all related with EGCG hepatotoxicity. EGCG also elevated interleukin-6 and monocyte chemoattractant protein-1 levels in the blood [355]. A series of tests were conducted to determine the toxicity of purified green tea extracts with high concentrations of EGCG to determine the safety of Teavigo, a high-concentration EGCG extract. Topical administration of EGCG extract had minor dermal irritation properties in rats and guinea pigs but not rabbits. In the guinea pig maximization test, topical EGCG formulations generated modest cutaneous irritation. An eye irritation test on rabbits revealed a strong enough response to rule out further testing in this assay. An oral dose of a 2000 mg EGCG preparation/kg killed rats, whereas a dose of 200 mg EGCG/kg had no adverse effects. At doses of up to 500 mg/kg/day, food feeding of an EGCG preparation to rats for 13 weeks was not hazardous. Similarly, when a 500 mg EGCG preparation/kg/day was delivered in divided doses to pre-fed dogs, no adverse effects were seen. When administered to starved dogs as a single bolus dosage, this dose induced morbidity, although this model was regarded as an impractical analogy to human consumption. These trials established a non-observed adverse impact limit of 500 mg of EGCG preparation/kg/day [356]. Similarly, following oral dosage using capsules, a standardized green tea extract was tested for exposure and toxicity in beagles. EGCG was the major component of the preparation, accounting for 56–72% of the material. To take advantage of the reported enhanced catechin bioavailability with fasting, a 9-month chronic trial (0, 200, 500, and 1000 mg/kg/day) was conducted in fasted dogs. Extensive morbidity, mortality, and pathology of several major organs resulted in its premature conclusion at 6.5 months, preventing the elucidation of the toxicity reasons. A 13-week follow-up study looked at the extract’s toxicity and exposure. The toxicities were generally less severe than in the chronic study at the same time. Dosing in a fed state resulted in significantly lower and less varied exposure than in fasted settings. Toxicity was less common and less severe with reduced exposure, but the small sample size and high variability precluded a definitive conclusion from being reached [357]. Micronuclei production in bone marrow cells was not induced by oral administration of 500, 1000, or 2000 mg EGCG/kg to mice. Similarly, exposing them to 400, 800, or 1200 mg EGCG/kg/day for 10 days did not induce the bone marrow cell micronuclei while generating plasma EGCG concentrations comparable to those seen in human investigations. The intravenous administration of 10, 25, and 50 mg EGCG/kg/day to rats resulted in significantly greater plasma concentrations and the absence of EGCG’s genotoxic effects. According to the findings of these investigations, Teavigo (EGCG) is not genotoxic [358]. Feeding pregnant rats meals supplemented with 1400, 4200, or 14,000 ppm throughout organogenesis was not hazardous to dams or fetuses in a major teratogenicity investigation. A two-generation study in rats administered with EGCG preparations at 1200, 3600, or 12,000 ppm found no negative effects on reproduction or fertility. The greatest dose lowered the progeny growth rate and caused a modest increase in pup loss. At 3600 ppm, there was also a growth effect in pups, but this was only observed in the second generation. The lowest dose was determined to be the overall non-observed adverse effect level (NOAEL). The NOAEL was similar to the 200 mg/kg/day EGCG preparation because the dams consumed twice as much feed during the critical lactation period [359]. A review of adverse event (AE) data from 159 human intervention studies found that only a few kinds of concentrated catechin-rich green tea preparations caused hepatic AEs in a dose-dependent manner when consumed in large bolus doses but not when utilized as brewed tea or extracts in beverages or as part of food. Toxico- and pharmacokinetic research indicates that the internal dose of catechins is a critical variable in the prevalence and severity of hepatotoxicity. Toxological and human safety data for tea preparations consumed as a solid bolus dose were used to calculate a safe intake level of 338 mg of EGCG/day for adults. Based on human AE data, an observed safe level (OSL) of 704 mg of EGCG/day could be proposed for tea preparations in a drinkable form [360].

Nevertheless, it is crucial to understand the fate of EGCG following this route of administration. The stomach’s acid (pH < 3) ensures the stabilization of EGCG following oral ingestion. The bioactivities and health effects of EGCG rely on its absorption and metabolism in the intestine. The small intestine absorbs some EGCG; however, a major fraction of EGCG passes from the small to the large intestine, where enterocytes and microbes convert it into up to eleven catechin ring-fission products [350]. EGCG is transported across the epithelium via passive diffusion, which explains the poor absorption of this catechin. Moreover, the compound may act as a substrate for multiple efflux transporters including multidrug-resistance-associated protein (MRP) efflux pumps [361] and P-glycoprotein (P-gp) [362], which limit the cellular uptake of the phenolic compound. This topic was previously reviewed [348,363].

As evidenced by clinical studies, the significant degree of variability in the pharmacokinetic parameters of EGCG might be explained by genetic variations in the genes encoding the drug transporters MRP2 and organic anion transporter SLC21A6 (OATP1B1). It is possible that this interindividual heterogeneity could account for the quantities of green tea polyphenols in human plasma or their potential to induce beneficial effects [364] and their potential to trigger pharmacokinetic interaction with other drugs [365,366,367,368].

**Figure 5:**
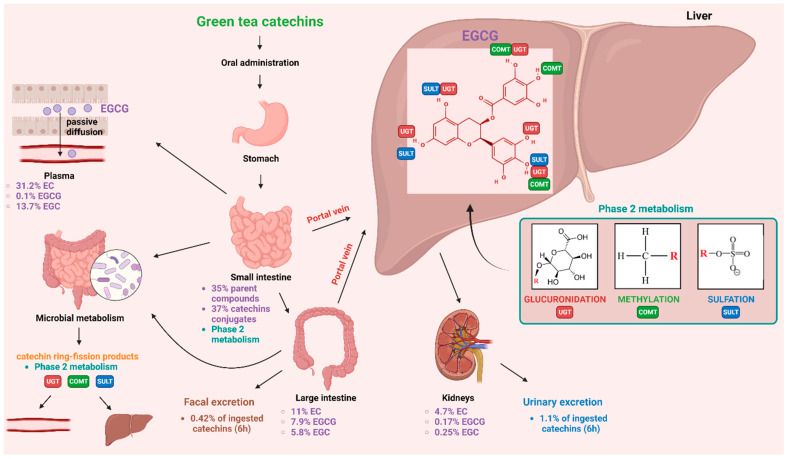
Overview of EGCG’s metabolism. Based on [347,348,372,373,374]. A detailed description can be found in the main text of the article. For full names, see the abbreviations section. Created with BioRender.com, accessed on 29 March 2023.

EGCG is susceptible to degradation or oxidation, thus conferring low EGCG concentrations in peripheral blood. Thereby, human plasma contains free and conjugated intestinal-microbiota-produced EGCG ring-fission metabolites [350]. Phase II enzymes, including UDP-glucuronosyltransferases (UGTs), sulphotransferases (SULTs), and catechol-O-methyltransferase (COMT), are responsible for the extensive metabolism of a portion of the ingested tea catechins before and after absorption, which occurs primarily in the small intestine and the liver [369]. Following ingestion, EGCG reaches a maximal plasma concentration after 90 min and is removed from the organism in the following 24 h [350]. Another study suggested that the elimination half-life of this phenolic compound is between 3.4 ± 0.3 h [370]. EGCG has been the subject of several more studies in both animal and human subjects aimed at assessing the bioavailability of this compound, some of which have shown conflicting findings [351,371]. An overview of EGCG’s metabolism is presented in Figure 5.

There is also no general agreement on the optimal level of EGCG that should be present in the human serum to achieve the greatest possible therapeutic benefits [350]. Several in vitro investigations have found that concentrations between 1–200 µM of EGCG are optimal for achieving the desired biological effects. Several pharmacokinetics investigations have shown that only a tiny percentage of unmodified EGCG reaches the bloodstream or tissues, making it challenging to achieve this effective concentration in vivo or in clinical research [347].

Recent investigations suggest the superior bioavailability of Teavigo^®^ (Healthy Origins, Pittsburgh, PA, USA) (capsules made from a refined green tea extract that have an EGCG level of around 94% with a greater solubility and stability compared to non-formulated EGCG extracts) compared to other available formulations such as FontUp^®^ (Grand Fontaine Laboratories, Barcelona, Spain) (Teavigo^®^ formulation combined with fats, carbohydrates, proteins, vitamins, and minerals). Moreover, the greatest bioavailability was achieved when the formulated extract was administered following fasting conditions (overnight) [350]. This is consistent with the earlier reports of EGCG’s bioavailability [375]. In contrast, taking EGCG in combination with dietary supplements (FontUp^®^) increased the molecule’s stability in the body [350]. The bioavailability and stability of EGCG have also been investigated in combination with other dietary components such as quercetin [376] or vitamin C [377]. Lazzeroni et al. investigated the effects of a lecithin formulation of a caffeine-free green tea catechin extract (Greenselect Phytosome (GSP)) on EGCG tissue distribution and its effect on cell proliferation and circulating biomarkers in breast cancer patients. Oral GSP was found to increase the bioavailability of EGCG [378].

Interesting clinical studies were performed to investigate whether or not the combined effects of drinking green tea and bathing in hot springs can lead to an increase in the yields of catechins that are absorbed into the body. The study compared two different interventions: drinking green tea while also taking a bath in a hot spring and drinking green tea on its own. Four healthy individuals had their plasma levels of EGCG measured. It was discovered that the combined practice of drinking green tea and taking a bath in hot springs resulted in a higher concentration of EGCG in the plasma [379].

### 4.4. Other Concerns

Despite the fact that the bioavailability of EGCG can strongly impact its conversion for clinical use, several other concerns need to be addressed:

- Standardization: Clinical research outcomes are difficult to evaluate and duplicate because of variations in the quality and purity of the EGCG preparations used.
- Safety concerns: Even though EGCG is usually believed to be safe, it might lead to liver damage in high doses, and there is a possibility that it could interact with some drugs. Additional research is required to completely understand the safety profile of this compound.
- Lack of clinical data: Although there is some evidence from preclinical research to show that EGCG may have a cancer-preventative effect, there is very little evidence from clinical studies that support its use in humans. More clinical tests that are carefully designed are required to assess the effectiveness and safety of this flavonoid.

## 5. Conclusions

**Figure 6:**
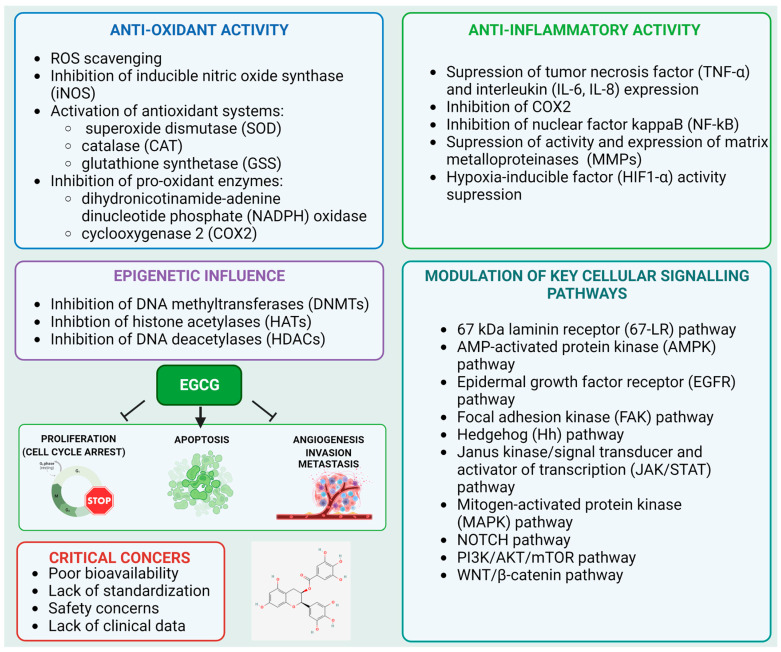
Anticancer properties of EGCG. EGCG exhibits antioxidant, anti-inflammatory, anti-proliferative, and anti-metastatic properties through the inhibition or modulation of key signaling pathways in cells and targets of epigenetic machinery. Details can be found in the main text of this article. Based on [94]. Created with BioRender.com, accessed on 29 March 2023.

EGCG, the main bioactive constituent of green tea, is a promising natural anti-cancer agent with a wide spectrum of health benefits. This phytochemical was shown to inhibit the growth, proliferation, and metastasis and trigger the apoptosis of multiple cancer cells derived from different tissues and organs in both in vitro and in vivo settings. The understanding of the molecular mechanisms of the anticancer activity of this compound revealed the versatile capability of this catechin to influence multiple pivotal signaling pathways inside the cell including, but not limited to, EGFR, JAK/STAT, PI3K/AKT/mTOR, and MAPK. EGCG has been found to modulate epigenetic mechanisms such as DNA methylation and histone modifications by targeting epigenetic modulators such as DNMTs, HATs, and HDACs (Figure 6).

Moreover, new emerging targets of EGCG have been identified. For example, EGCG acts as an ATP-competitive inhibitor of GRP78, a chaperone protein that promotes chemoresistance and inhibits apoptosis in cancer cells. EGCG has been found to suppress GRP78 activity and hinder its expression, particularly in glioblastomas. EGCG has been identified as a compound with a significant capability of HK2 inhibition. HK2 plays a role in the metabolic shift towards aerobic glycolysis in cancer cells. EGCG effectively suppresses ZAP-70 activity, a protein tyrosine kinase involved in the signaling process mediated by T-cell receptors. Through reverse docking methodology and network pharmacology, EGCG has been found to impact multiple signaling pathways and proteins. These include FYN, NOS2, CDK2, ABL1, SYK, AKT1/2, MAPK8, IRAK4, and APAF1, which have been previously investigated in in vitro studies. Additionally, novel targets including IKKβ, KRAS, NTRK1, and WEE1 have been identified. EGCG has shown a moderate suppression of WEE1 activity and an efficient inhibition of IKKβ, KRAS, and NTRK1 in enzymatic assays. Future studies may also elucidate new mechanisms through which EGCG may suppress the development of tumors or restrict their growth.

Clinical trial results from the last ten years suggest that EGCG may have beneficial effects on several health conditions. These conditions include amyloidosis, atherosclerosis, cystic fibrosis, Barrett’s esophagus, diabetes, Down’s syndrome, dystrophic epidermolysis bullosa, hypertension, multiple sclerosis, obesity, radiation-induced esophagitis, and ulcerative colitis. EGCG has shown anticancer activities against bladder cancer. It blocks the expression of purinergic receptors in urothelial cells and inhibits the release of ATP from those cells. Additionally, EGCG has demonstrated anti-inflammatory and antioxidant activity, as it inhibits the induction of iNOS and phosphorylated NF-κB. Some preclinical studies, epidemiologic studies, and previous clinical trials suggest that catechins, including EGCG, found in green tea may reduce the risk of developing prostate cancer. However, a clinical trial with a decaffeinated catechin mixture containing 400 mg of EGCG per day did not diminish the risk of prostate cancer in men with HGPIN and/or ASAP. The administration of GTE capsules containing 800 mg of EGCG seems to regulate biomarkers related to colorectal cancer pathogenesis, including selenoproteins, WNT signaling (β-catenin), inflammation (NF-κB), and methylation (DNMT1). A phase IB study conducted in patients with APL of the oral cavity and larynx showed that a combination of green tea polyphenon E (PPE) and erlotinib prolonged cancer-free survival. This combination was well tolerated, suggesting its potential as a chemopreventive measure for recurrent primary tumors in early-stage head and neck cancer patients.

Despite the initial enthusiasm for EGCG use in cancer treatment, conflicting epidemiological data were obtained considering EGCG consumption and the decreased risk of many cancers. No clear evidence suggests that tea consumption reduces the overall risk of developing cancer.

Furthermore, the clinical use of EGCG is hindered by its poor oral bioavailability, low intestinal absorption, and short retention time. Overcoming these challenges is necessary for its widespread use in clinical settings. Various approaches, such as structural alterations, nano-carriers, lipid carriers, and protein carriers, have been employed to improve the pharmacokinetics and pharmacological properties of EGCG, increasing its bioavailability and dosing interval. Moreover, the effects of nutrients, timing, and fasting were investigated to increase the intestinal absorption and bioavailability of EGCG. Additionally, the wide-spread access to EGCG and EGCG sources increases the toxicities in humans when its use is abused. Despite the fact that EGCG is usually believed to be safe, it might lead to liver damage in high doses, and there is a possibility that it could interact with some drugs. Additional research is required to completely understand the safety profile of this compound and establish an optimal dosage that will provide the most health benefits and decrease the chance of developing toxicity effects.

Although there is some evidence from preclinical research to show that EGCG may have a cancer-preventative effect, there is very little evidence from clinical studies that supports its use in humans. More clinical tests that are carefully designed are required to assess the effectiveness and safety of this compound. Clinical research outcomes are often difficult to evaluate and duplicate because of variations in the quality and purity of EGCG preparations used. Therefore, standardization is required.

## Figures and Tables

**Table 1:** Ongoing and future clinical studies on EGCG in cancer treatment.

Title	Number	Status	Estimated Enrollment	Objective
A Pilot Study to Evaluate the Chemopreventive Effects of Epigallocatechin Gallate (EGCG) in Colorectal Cancer (CRC) Patients with Curative Resections.	NCT02891538	Recruiting	50	Evaluating cthe hemopreventive properties of EGCG in CRC patients with curative resections by comparing DNA methylation change during 1 year of EGCG treatment.
The Study of Quadruple Therapy Quercetin, Zinc, Metformin, and EGCG as Adjuvant Therapy for Early, Metastatic Breast Cancer and Triple-negative Breast Cancer, a Novel Mechanism	NCT05680662	Not yet recruiting	200	Achieving the best efficacy for different stages breast cancer and triple-negative breast cancer treatment in female patients by applying various combinations of adjuvants.
Study of Epigallocatechin-3-gallate (EGCG) for Supportive and Symptomatic Management in Patients with Esophageal Cancer	NCT05039983	Recruiting	15	Assessing the application of EGCG for alleviating esophageal stenosis, choking and pain while swallowing, weight change, and blood parameter changes in pathological esophageal squamous cell carcinoma patients.
Reducing Frailty for Older Cancer Survivors Using Supplements (ReFOCUS): A Phase 2 Randomized Controlled Trial of Epigallocatechin-3-Gallate (EGCG) on Frailty and Inflammation in Older Survivors of Cancer	NCT04553666	Recruiting	40	Investigating the beneficial properties and safety of EGCG treatment in older cancer survivors with frailty and inflammation.
A Phase II Randomized Double Blinded Study of Green Tea Catechins (GTC) vs. Placebo in Men on Active Surveillance for Prostate Cancer: Modulation of Biological and Clinical Intermediate Biomarkers	NCT04597359	Recruiting	360	Assessing the abilities of tea catechins in preventing the progression of prostate cancer using various genetic, biochemical, and clinical markers.
Phase II Clinical Trial of Green Tea Catechins in Men on Active Surveillance (AS)	NCT04300855	Recruiting	135	An evaluation of the bioavailability, safety, effectiveness, and validation of the acting mechanism of a drug containing EGCG applied for 24 months to men with adenocarcinoma of the prostate with cancer present.
Pioneering Pre- and Post-Operative Integrative Care to Improve Thoracic Cancer Quality of Care—The Thoracic Peri-Operative Integrative Surgical Care Evaluation (POISE) Trial—Stage II	NCT04871412	Recruiting	20	Improving health-related quality of life, decreasing surgical adverse events, prolonging overall survival, and pioneering integrative care delivery for thoracic cancer patients.
Fibroids and Unexplained Infertility Treatment With Epigallocatechin Gallate; A Natural CompounD in Green Tea (FRIEND)	NCT05364008	Recruiting	200	Determining the effect of low-caffeine green tea extract containing 45% EGCG on fibroids and subsequent pregnancy and live births in women seeking fertility treatment.
The Use of Vitamin D in Combination With Epigallocatechin Gallate, D-chiro-inositol and Vitamin B6 in the Treatment of Women With Uterine Fibroid	NCT05448365	Recruiting	60	Evaluating the impact on uterine fibroid volume of a combination of natural molecules including epigallocatechin gallate, vitamin D, D-chiro-inositol, and vitamin B6.
Effects of Vitamin D, Epigallocatechin Gallate, Vitamin B6, and D-Chiro-inositol Combination on Uterine Fibroids: a Randomized Controlled Trial	NCT05409872	Recruiting	108	Evaluating the efficacy of a combination of epigallocatechin gallate, vitamin D3, D-chiro-inositol, and vitamin B6 as a treatment for uterine fibroids.
A Phase I Single-arm, Multicenter Pilot Study Aimed at Validating γ-OHPdG as a Biomarker and Testing the Effects of Polyphenon E on Its Levels in Patients With Cirrhosis	NCT03278925	Active, not recruiting	48	Investigating the side effects and best dose of defined green tea catechin extract and verifying its ability to prevent liver cancer in participants with cirrhosis.
A Pilot Study of Gemcitabine, Abraxane, Metformin and a Standardized Dietary Supplement (DS) in Patients With Unresectable Pancreatic Cancer	NCT02336087	Active, not recruiting	21	Investigating the side effects of gemcitabine hydrochloride, nab-paclitaxel, metformin hydrochloride, and a standardized dietary supplement including EGCG in treating patients with pancreatic cancer that cannot be removed by surgery.
Phase ⅠStudy of Oral Green Tea Extract as Maintenance Therapy for Extensive-stage Small Cell Lung Cancer	NCT01317953	Available	No information	Assessing the safety of the application of EGCG for extensive-stage small lung cancer patients who have achieved an objective tumor response after first-line therapy.

## Data Availability

No new data were created.

## Abbreviations

4E-BP1	Eukaryotic-translation-initiation-factor-4E-binding protein 1
67LR	67-kD laminin receptor
ABL1	Tyrosine-protein kinase ABL1
AIF	Apoptosis-inducing factor
AKT	Protein kinase B
AP-1	Activator protein-1
APAF1	Apoptotic-protease-activating factor 1
APL	Acute promyelocytic leukemia
AR	Androgen receptor
ASAP	Atypical small acinar proliferation
ASM	Acid sphingomyelinase
BAX	Bcl-2-associated X protein
BCL-2	B-cell lymphoma 2
BCL-xL	B-cell lymphoma-extra large
BCR	B-cell receptor
CAFs	Cancer-associated fibroblasts
CD3	Cluster of differentiation 3
CDH1	Cadherin 1
CDK2	Cyclin-dependent kinase 2
CDK4/6	Cyclin-dependent kinases 4/6
c-IAP1/2	Cellular inhibitor of apoptosis protein 1/2
CLL	Chronic lymphocytic leukemia
COMT	Catechol-O-methyltransferase
COX-1	Cyclooxygenase-1
COX-2	Cyclooxygenase-2
COXs	Cyclooxygenases
CpG	Cytosine-phosphate-guanine
DAPK1	Death-associated protein kinase 1
DBGT	Double-brewed green tea
DNA-PK	DNA-dependent protein kinase
DNMT	DNA methyltransferase
DR5	Death receptor 5
XIAP	E3 ubiquitin-protein-ligase-X-linked inhibitor of apoptosis protein
EC	Epicatechin
ECG	Epicatechin-3-gallate
ECM	Extracellular matrix
EGC	Epigallocatechin
EGCG	Epigallocatechin-3-gallate
EGF	Epidermal growth factor
EGFR	Epidermal growth factor receptor
ER	Endoplasmic reticulum
ERK1/2	Extracellular-signal-regulated kinases 1 and 2
ETC	Electron transport chain
EZH2	Enhancer of zeste homolog 2
FAK	Focal adhesion kinase
FAS	Fatty acid synthase
FLIP	Cellular FLICE-like inhibitory protein
GADD153	Growth arrest and DNA-damage-inducible gene 153
GLI1	GLI-Kruppel family member 1
GLUTs	Glucose transporters
GRP78	Glucose-regulated protein 78
GST P1-1	Glutathione S-transferase P1-1
GTE	Green tea extract
HDAC	Histone deacetylase
HER-2	Human epidermal growth factor receptor 2
HGF	Hepatocyte growth factor
HGPIN	High-grade prostatic intraepithelial neoplasia
Hh	Hedgehog
HIF-1α	Hypoxia-inducible factor 1-alpha
HK2	Hexokinase 2
HUVECs	Human umbilical vein endothelial cells
ICAM-1	Intercellular adhesion molecule-1
IFNs	Interferons
IGF-1	Insulin-like growth factor 1
IGFBP-3	IGF-binding protein-3
IGF-1R	Insulin-like growth factor-1 receptor
IKKβ	Inhibitor of nuclear factor kappa-B kinase subunit beta
IL-1β	Interleukin 1β
IL-2	Interleukin 2
IL-4	Interleukin 4
IL-6	Interleukin 6
IL-8	Interleukin 8
iNOS	Inducible nitric oxide synthase
IRAK4	Interleukin-1-receptor-associated kinase 4
IRF1	Interferon regulatory factor 1
JAK	Tyrosine-protein kinase JAK
JNK	c-Jun N-terminal kinase
Ki-67	Antigen Ki-67
KRAS	GTPase KRas
MAPK8	Mitogen-activated protein kinase 8
MCP-1	Monocyte chemoattractant protein-1
MD	Molecular dynamics
MMPs	Matrix metalloproteinases
MRP	Multidrug-resistance-associated protein
mTOR	Serine/threonine-protein kinase mTOR
NADPH	Nicotinamide adenine dinucleotide phosphate
NF-Kb	Nuclear factor kappa-light-chain-enhancer of activated B cells
NO	Nitric oxide
NOS	Nitric oxide synthase
NOTCH	Neurogenic locus notch homolog protein
NPs	Natural products
NSCLC	Non-small-cell lung cancer
NTRK1	High-affinity nerve growth factor receptor
OATP1B1	Organic anion-transporting polypeptide 1B1
P300/CBP	CREB-binding protein/p300
PARP	Poly (ADP-ribose) polymerase
PcG	Polycomb group
PDK1	Phosphoinositide-dependent kinase-1
PD-L1/2	Programmed death-ligand 1/2
PFK	Phosphofructokinase
PFKP	Platelet-type phosphofructokinase
PGE2	Prostaglandin E2
P-gp	P-glycoprotein
PI3K	Phosphatidylinositol-3-kinase
PIP2	Phosphatidylinositol 4,5-bisphosphate
PIP3	Phosphatidylinositol 3,4,5-triphosphate
PK	Pyruvate kinase
PKC	Protein kinase C
PKM2	Pyruvate kinase M2
PP2A	Protein phosphatase 2A
PPE	Green tea polyphenon E
PRC2	Polycomb-repressive complex 2
PSA	Prostate-specific antigen
PTEN	Phosphatidylinositol-3,4,5-trisphosphate 3-phosphatase and dual-specificity protein phosphatase
RARβ	Retinoic acid receptor beta
RAS/MAPK	RAS/mitogen-activated protein kinase
ROS	Reactive oxygen species
S6K	Ribosomal protein kinase S6
SAM	S-adenosylmethionine
SHH	Sonic hedgehog
SIRT6	Sirtuin-6
SLC21A6	Organic anion transporter
SMO	Smoothened
SPR	Surface plasmon resonance
STAT	Signal transducer and activator of transcription
SULTs	Sulphotransferases
SYK	Spleen tyrosine kinase
TAMs	Tumor-associated macrophages
TCR	T-cell receptor
TIMP-3	Tissue inhibitor of matrix metalloproteinase-3
TLR4	Toll-like receptor 4
TME	Tumor microenvironment
TNFR-1/2	TNF receptor 1/2
TNF-α	Tumor necrosis factor α
TP53	Tumor protein p53
UGTs	UDP-glucuronosyltransferases
VEGF	Vascular endothelial growth factor
VEGFR	Vascular endothelial growth factor receptor
WEE1	Wee1-like protein kinase
ZAP-70	Zeta-chain-associated protein kinase-70
γ-CD	γ-cyclodextrin
